# Tuning the Properties of Biobased PU Coatings via Selective Lignin Fractionation and Partial Depolymerization

**DOI:** 10.1021/acssuschemeng.3c00889

**Published:** 2023-04-21

**Authors:** Arjan T. Smit, Emanuela Bellinetto, Thomas Dezaire, Oussama Boumezgane, Luke A. Riddell, Stefano Turri, Michiel Hoek, Pieter C. A. Bruijnincx, Gianmarco Griffini

**Affiliations:** † The Netherlands Organisation for Applied Scientific Research (TNO), unit Energy Transition, Biobased & Circular Technologies group, P.O. Box 1, 1755 ZG Petten, The Netherlands; ‡ Department of Chemistry, Materials and Chemical Engineering “Giulio Natta”, Politecnico di Milano, Piazza Leonardo da Vinci 32, 20133 Milano, Italy; § Organic Chemistry and Catalysis, Institute for Sustainable and Circular Chemistry, Utrecht University, Universiteitsweg 99, 3584 CG Utrecht, The Netherlands

**Keywords:** biomass organosolv pretreatment, lignin fractionation, reductive depolymerization, tailored lignin molar mass and reactivity, tunable PU coating properties

## Abstract

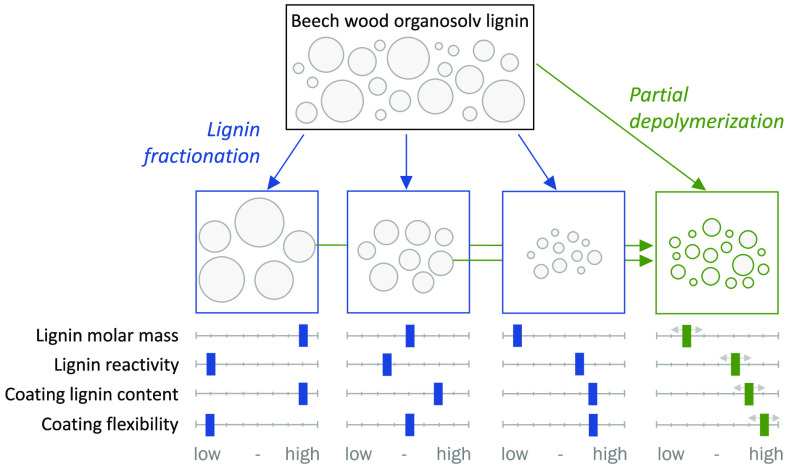

Polyurethane (PU) coatings with high lignin content and tunable properties were made using a combination of fractionation and partial catalytic depolymerization as a novel strategy to tailor lignin molar mass and hydroxyl group reactivity, the key parameters for use in PU coatings. Acetone organosolv lignin obtained from pilot-scale fractionation of beech wood chips was processed at the kilogram scale to produce lignin fractions with specific molar mass ranges (*M*_w_ 1000–6000 g/mol) and reduced polydispersity. Aliphatic hydroxyl groups were distributed relatively evenly over the lignin fractions, allowing detailed study of the correlation between lignin molar mass and hydroxyl group reactivity using an aliphatic polyisocyanate linker. As expected, the high molar mass fractions exhibited low cross-linking reactivity, yielding rigid coatings with a high glass transition temperature (*T*_g_). The lower *M*_w_ fractions showed increased lignin reactivity, extent of cross-linking, and gave coatings with enhanced flexibility and lower *T*_g_. Lignin properties could be further tailored by lignin partial depolymerization by reduction (PDR) of the beech wood lignin and its high molar mass fractions; excellent translation of the PDR process was observed from laboratory to the pilot scale necessary for coating applications in prospective industrial scenarios. Lignin depolymerization significantly improved lignin reactivity, and coatings produced from PDR lignin showed the lowest *T*_g_ values and highest coating flexibility. Overall, this study provides a powerful strategy for the production of PU coatings with tailored properties and high (>90%) biomass content, paving the path to the development of fully green and circular PU materials.

## Abstract 2

Lignin fractionation and partial depolymerization provide a powerful strategy for the production of biobased PU coatings with tailored properties.

## Introduction

Polyurethanes (PUs) are a versatile class of polymers, generally consisting of petrochemical polyols (having two or more hydroxyl groups) and isocyanates (having two or more isocyanate groups) connected via a urethane linkage. Typically, the polyol (e.g., 1,4-butanediol, poly(ethylene glycol)) is the component that enables tuning of the mechanical properties of the product, e.g., partly controlling product rigidity and flexibility by using short- or long-chain polyols, respectively. Further product property control can be obtained by selection of the polyisocyanate cross-linker, with aromatic isocyanates (e.g., toluene diisocyanate and 4,4′-methylene diphenyl diisocyanate) providing more rigidity and aliphatic isocyanates (e.g., hexamethylene diisocyanate) increasing the product flexibility.^1^ Overall, the wide range of accessible PU product characteristics provides access to a variety of high-value applications such as foams, coatings, elastomers, and adhesives.^2^ Lignin, an abundantly available biopolymer isolated from lignocellulosic biomass via various pretreatment processes, is widely researched as a sustainable and green alternative to the petrochemical PU building blocks, the polyols in particular.^3^ Indeed, due to its aromatic structure and relatively high hydroxyl group (OH) density, lignin has shown potential as a renewable polyol for the production of biobased coatings and resins with enhanced degree of cross-linking and improved mechanical and thermal properties of the product.^4−9^ To minimize production cost and maximize sustainability impact, biobased PU coatings are preferably produced from unmodified lignin. However, the heterogeneity of the lignin structure, in particular its high polydispersity, and the often insufficient solubility of lignin in conventional solvents are key challenges that restrict its direct use in high-value applications such as PU coatings. Additionally, some of the lignin hydroxyl groups lack reactivity, being located inside the lignin macromolecular structure where steric hindrance impairs accessibility to isocyanate.^10^

Previously reported work from some of us detailed the reaction of a vanillic acid-derived diisocyanate with beech wood acetone organosolv lignin, kraft softwood lignin, and wheat straw soda lignin for the production of thermosetting PU coatings with a high biomass content.^11^ The organosolv lignin showed limited aliphatic hydroxyl group reactivity, likely caused by its relatively high molar mass. The high organosolv lignin content of the PU coating preparations and limited degree of cross-linking affected the viscoelastic behavior of the coatings, showing larger creep deformation and lower creep recovery as compared to the kraft and soda lignin-based coatings.^11^

Consequently, tailoring of the reactivity and physicochemical properties of lignins is the way forward to arrive at PU coatings with high lignin content, tailored characteristics, and superior quality. For example, lignin reactivity may be improved by applying chemical functionalization routes to convert the relatively unreactive phenolic hydroxyl groups to more reactive aliphatic hydroxyl groups.^5,12−16^ Extending the aliphatic groups outside of the lignin macromolecular structure may reduce steric hindrance and further improve reactivity. However, chemical modification of as-isolated lignins does not address the issue of lignin molar mass heterogeneity.

Fractionation of lignin into lower dispersity fractions of varying molar mass is a particularly attractive and practical strategy to deal with the challenge of lignin molecular weight heterogeneity.^17^ Different lignin fractionation approaches have been applied on technical lignins (i.e., lignins isolated after biomass pretreatment) with lignin fractions been obtained through membrane filtration,^18,19^ selective precipitation,^20−22^ and sequential solubilization in a variety of solvents.^23,24^ In addition to having a better-defined molecular weight, lignin fractions also typically vary in hydroxyl group content. A Kraft lignin fraction with lower molecular weight and a higher OH content was reported to show improved reactivity properties and resulted in improved coating characteristics.^25,26^

An alternative strategy to homogenize and tune the lignin properties is by depolymerization, reducing its molar mass and dispersity. Partially depolymerized lignin obtained from alkaline hydrothermal treatment reportedly enhanced lignin dispersion in the coating and improved its mechanical properties.^27,28^ Notably, how amenable a technical lignin is toward depolymerization is highly dependent on the severity of the pretreatment method used for isolation, as this determines how many labile ether bonds remain in the lignin as well as the degree of condensation through C–C bond formation, the bonds that are more recalcitrant and less prone to cleavage.

Acetone organosolv fractionation, the so-called Fabiola process, enables the isolation of sugars and lignin in relatively high yield and purity from a wide variety of lignocellulosic biomass streams.^29,30^

The relatively mild pretreatment process preserves part of the cleavable ether bonds and thus produces a lignin that is less-condensed as compared to other technical lignins. The organosolv process was validated at pilot scale using industrial size beech wood chips, low liquid-to-solid ratios, and continuous lignin precipitation from the pulping liquor.^31^ The structural characteristics of the isolated lignin are similar to the lignin used in the aforementioned study with a vanillic acid-derived diisocyanate.^11^

**Figure 1:**
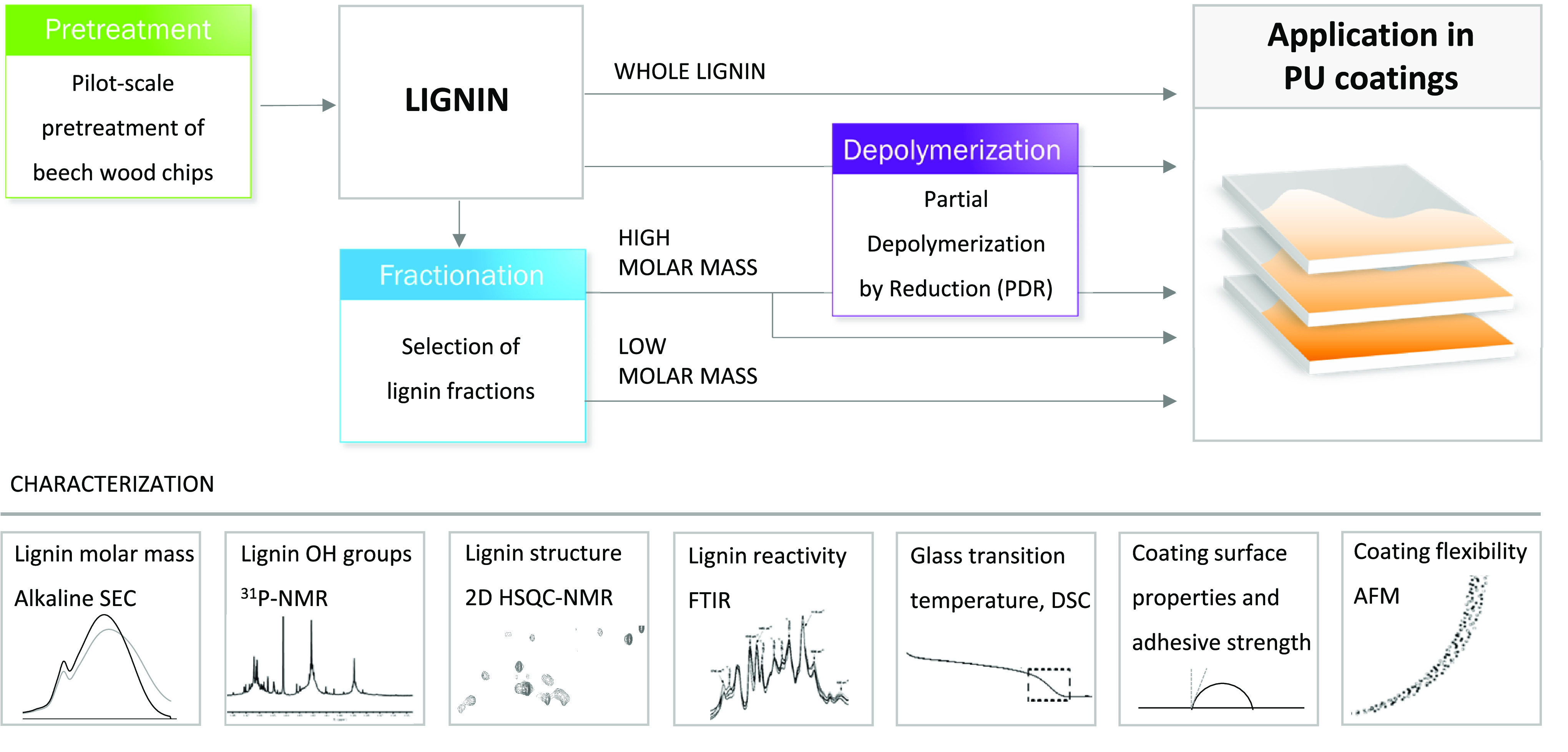
Experimental design for lignin fractionation, partial depolymerization, and application of the fractions in PU coatings.

To further improve the use of acetone organosolv lignin in PU coatings and introduce tunable coating characteristics, a new strategy is presented here (Figure 1) that aims to control lignin homogeneity and to tailor lignin molar mass and dispersity, thereby improving lignin solubility and hydroxyl group reactivity. Beech wood lignin fractions, with molar masses ranging from roughly 1000 to 6000 g/mol, were obtained by stepwise precipitation of beech wood lignin from 60% aqueous acetone, gradually increasing the amount of water as antisolvent, similar to the earlier reports by Sadeghifar et al. and Jääskeläinen et al.^21,32^ The as-is beech wood lignin and selected fractions are used as polyol for the formation of PU coatings. Additionally, the recently reported strategy for lignin partial depolymerization by reduction (PDR),^33^ which aims to reduce lignin molar mass and dispersity by cleaving the remaining lignin β-aryl ethers using reductive conditions, was applied to the organosolv beech wood lignin and one of its high-molar-mass lignin fractions. PDR was successfully upscaled to the kilogram lignin scale to produce depolymerized lignin in sufficient quantity for application testing.

The PU coatings are prepared using a classical aliphatic hexamethylene diisocyanate-based linker (HMDI), a component that evidently does not yet comply with the general sustainability criteria desired for biobased processes and products. However, studying the renewable lignin component with such an industrially relevant and widely employed cross-linker to produce our PU materials is needed to first provide a reliable reference test bed for establishing clear polyol structure–coating performance relationships and demonstrate a viable way to increase biobased content in industrially scalable and high-performance coating formulations.

Combining fractionation and PDR to address the challenge of lignin heterogeneity, we thus show that lignin-based PU coating characteristics can be improved and, importantly, tailored, presenting a promising strategy for the development of high-value lignin applications as well as providing support for further development of biobased isocyanate-free cross-linkers to arrive at fully green and circular PU coatings.

## Results and Discussion

### Lignin Isolation, Fractionation, and Fraction Selection

Beech wood acetone organosolv lignin (OSL) was produced at pilot scale, subjecting 70 kg of industrial-size beech wood chips to pretreatment in 50% aqueous acetone at 140 °C using a low liquid-to-solid ratio (L/S 3.3 based on dry wood). Lignin was precipitated from the pulping liquor using the LigniSep technology of a continuous falling film precipitator to enable the isolation of OSL lignin in high yield and purity, whose characteristics are detailed in a previous publication (as P-BEC-3).^31^ OSL was resolubilized in 60% aqueous acetone and precipitated in well-defined steps by addition of water, after which the precipitated lignin fractions were collected by centrifugation (see the Supporting Information for details). Water was added to obtain fractions at an acetone concentration of 45% (F1), 40% (F2), 30% (F3), and 15% (F4). Thereafter, the supernatant liquor was reduced to dryness to obtain the final solid fraction (F5).

**Figure 2:**
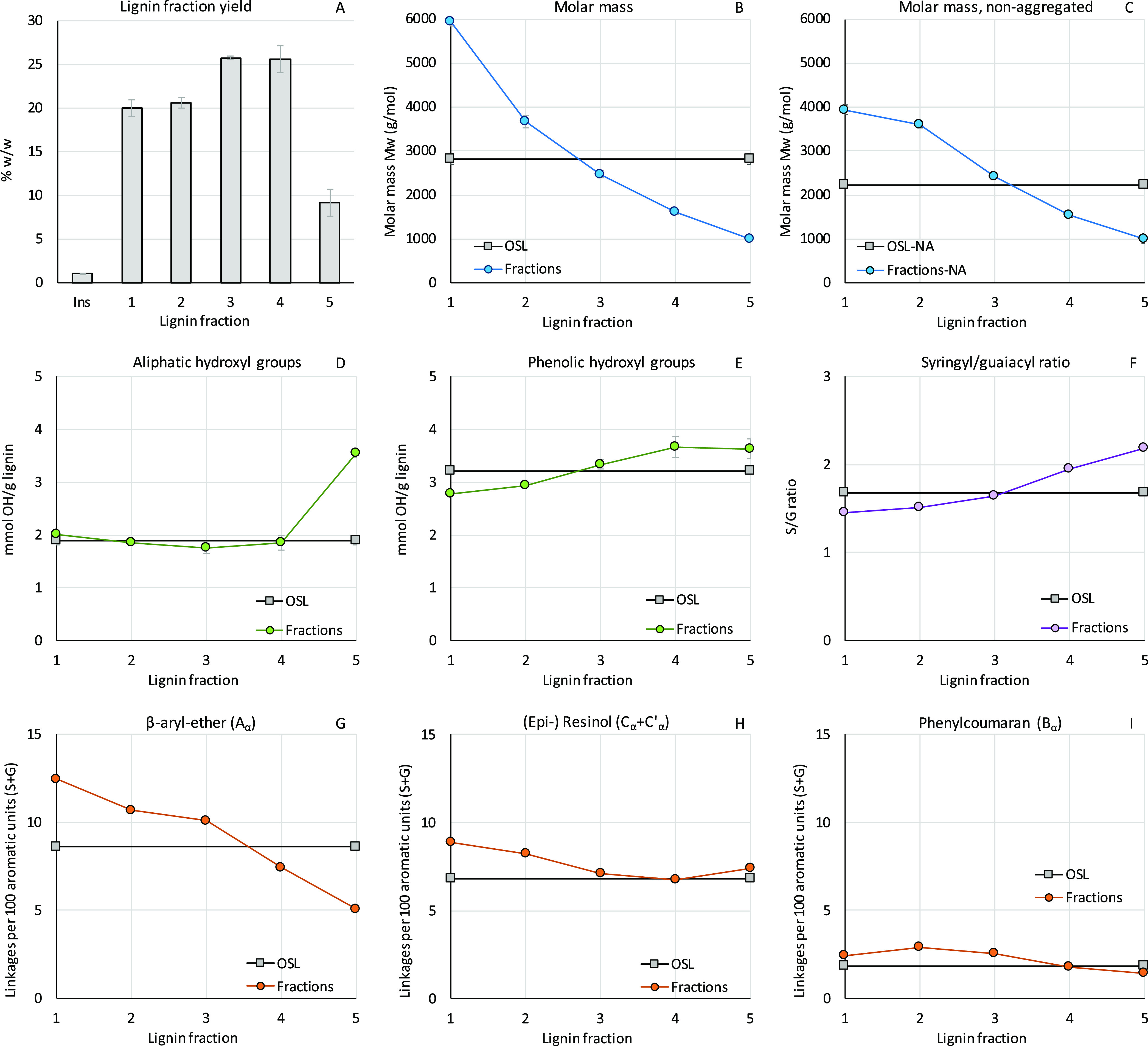
OSL fraction yield and characteristics of untreated OSL (gray squares) and OSL fractions (colored markers). (A) lignin fractionation yield, (B) weight average molecular weight of as-is and (C) nonaggregated OSL and fractions, (D) aliphatic and (E) phenolic hydroxyl groups, (F) syringyl/guaiacyl ratio, abundance of (G) lignin β-aryl ether linkages, (H) (epi-)resinol linkages, and (I) phenylcoumaran linkages of OSL and fractions.

As shown in the fraction distribution in Figure 2A, a small part of the lignin did not dissolve in 60% acetone (Ins, 1.0% w/w) and was excluded from the study. OSL contains both free and lignin-bound sugars (1% w/w in total), and in this particular fractionation method, the free sugars accumulate in F5 (7.7% w/w, Table S2). The lignin fractions were characterized by alkaline size exclusion chromatography (SEC) to determine the lignin molar mass and dispersity. As described in a previous work, lignin aggregates, likely formed during lignin precipitation, do not fully dissociate in the 0.5 M NaOH solution used for SEC analysis leading to an overestimation of the lignin molar mass.^33^

To resolve such an aggregation issue, the lignin was heated in methanol under an argon atmosphere (30 bar) to 150 °C, followed by immediate cooling and subsequent evaporation of the solvent to obtain a dry lignin sample that was subjected to SEC analysis. This procedure reduces aggregation, as reflected in the lower recorded lignin molar masses, without altering its structure or linkage abundance.^33^Figure 2B,C shows a significant effect of this treatment on OSL lignin, lowering its weight-average molecular weight (*M*_w_) from 2830 to 2230 g/mol. Surprisingly, lignin aggregation is apparently limited to (very) large lignin fragments, as only for F1 a large difference between aggregated and nonaggregated lignin is observed. As expected, lignin fractionation results in the isolation of larger lignin fragments at higher acetone concentrations (F1) followed by consecutively lower molar masses in the fractionation series.

**Figure 3:**
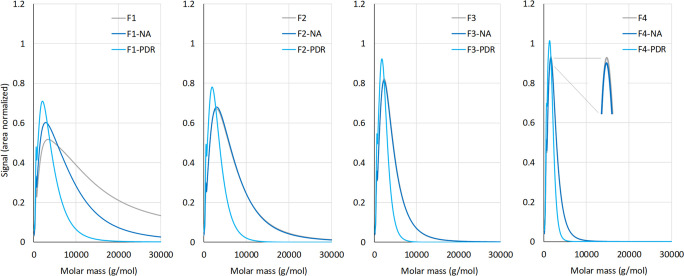
SEC curves of isolated, nonaggregated (-NA), and partially depolymerized (PDR) OSL fractions F1, F2, F3, and F4.

Note that the weighed sum of the fraction molar masses (i.e., summative molar masses corrected for the precipitation yield in each fraction) at 3090 and 2640 g/mol for aggregated and nonaggregated lignin is higher than that measured for as-is OSL (2830 and 2230 g/mol, respectively). Therefore, it is important to interpret the reported *M*_w_ values in conjunction with the SEC curves (presented in Figures 3, S2, and S7–S10).

Lignin hydroxyl group density as determined by ^31^P NMR (Figure 2D,E) shows an increase in lignin phenolic hydroxyl group (Ph-OH) content upon decreasing lignin molar mass, in accordance with similar lignin fractionation studies.^21,32,34^ Abundance of aliphatic hydroxyl groups (Al-OH) does not differ significantly for the lignin fractions except for the high abundance of Al-OH in F5, where the presence of sugars contributes to the Al-OH content. Contrary to the lignin molar mass, here, the weighed sum of fraction aliphatic and phenolic hydroxyls and the carboxylic acid groups corresponds well with the as-is OSL values (Table S2). 2D-HSQC NMR analysis of the lignin fractions (Figures 2G–I, S3, S4 and Table S2) revealed a slight increase in syringyl (S) units from F1 to F5. Interunit linkages such as β-aryl ether (β-O-4), and to a lesser extent resinol (β–β) and phenylcoumaran (β-5) generally get less abundant from F1 to F5. Overall, the distribution of lignin structural characteristics and the relation with lignin molar mass are similar to the THF–methanol–hexane fractionated CELF lignins reported by Wang et al.^35^ The assigned NMR spectra as well as distribution of minor structures over the lignin fractions are available in Table S2, Figures S3 and S4.

### Lignin Partial Depolymerization by Reduction (PDR)

**Figure 4:**
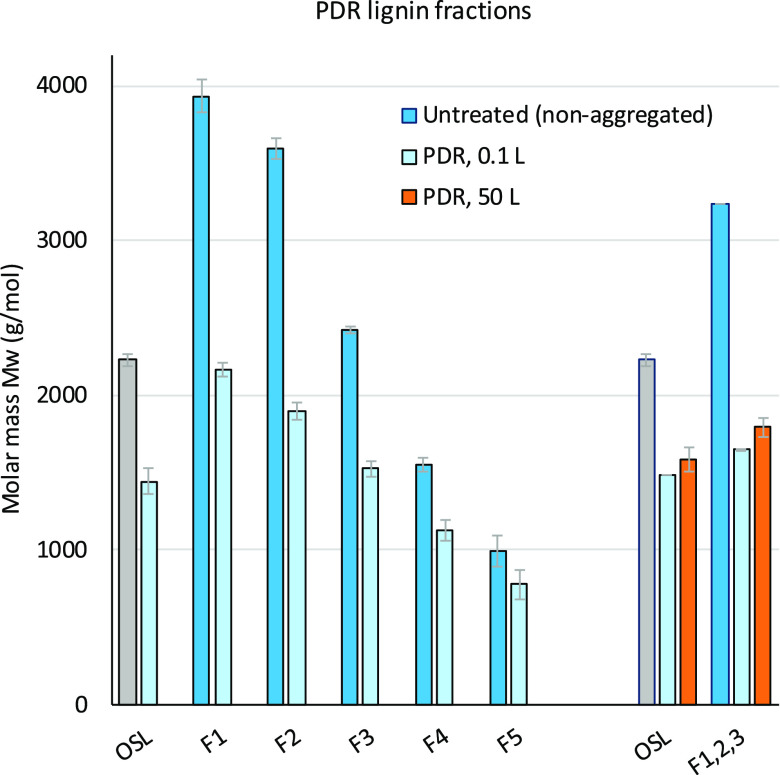
Molar mass of untreated and depolymerized OSL and its fractions.

The recently reported lignin PDR process aims to produce lignins with a lower molar mass and dispersity.^33^ Specifically for PU applications, the smaller lignin fragments are anticipated to combine better physicochemical properties with improved reactivity, i.e., higher accessibility for diisocyanates to the lignin hydroxyl groups, and thus increased extent of cross-linking. PDR was conducted using previously established optimized conditions that balance molar mass reduction with maintaining lignin aliphatic hydroxyl group content. Depolymerization for 2 h at 200 °C using 10 g/L lignin, 0.5 g of Ru/C per gram lignin, and 30 bar H_2_ proved best in balancing between partial depolymerization and preserving 4-*n*-propanol end group units (rather than generating 4-*n*-propyl end groups). Laboratory-scale PDR (0.1 L, Figure 4) of OSL resulted in a lignin molar mass decrease from 2230 to 1440 g/mol and drop in dispersity from 2.4 to 1.9. Depolymerization of the OSL fractions under identical conditions similarly showed a 24–50% reduction in molar mass; depolymerization was most extensive for the β-O-4 (A_α_)-rich F1 and F2 fractions, as expected. These fractions also showed the highest increase in phenolic OH content (resulting from β-O-4 cleavage) and, rewardingly, mostly unchanged lignin aliphatic OH content after partial depolymerization (Figure S11).

To produce sufficient partially depolymerized lignin for the coating application, the process was scaled-up using a 50 L batch reactor. The results of a run with 900 g of OSL and 450 g of Ru/C in 30 L MeOH under 20 bar hydrogen at 200 °C proved to be comparable to the laboratory-scale tests in terms of molar mass reduction (orange bar, Figure 4). While large-scale depolymerization did increase the lignin aliphatic hydroxyl group content (Table 1, S4), overall, the hydroxyl group content was slightly lower than that for the laboratory-scale PDR product (Figure S11). A scale-up experiment was also conducted using the combined higher molar mass fractions F1, F2, and F3. The mixture, corrected for lignin fraction yield, consisted of 30% F1, 30% F2, and 40% F3 (see SI for more details). Scale-up reaction conditions were adjusted to 2 h isothermal at 200 °C using 750 g of lignin and 375 g of Ru/C in 25 L methanol under 20 bar hydrogen pressure prior to heating. Here again, the resulting PDR lignin molar mass is comparable to the laboratory-scale one conducted under identical process conditions: molar mass was reduced from 3240 to 1790 g/mol and the aliphatic hydroxyl group content increased from 1.86 to 2.27 mmol/g lignin.

**Table 1:** Lignin Fractions and Compositional Characteristics of Lignin-Based PU Coatings

	lignin characteristics				coating composition		
	*M*_w_^a^ [g/mol]	polydispersity	aliphatic OH [mmol/g]	*T*_g_ (°C)	aliphatic OH/NCO molar ratio	lignin used [mass %]	*T*_g_ (°C)
OSL	2230 ± 40	2.4	1.89 ± 0.08	124	2.50	87	155
F1	3940 ± 110	4.2	2.01 ± 0.04	169	7.50	95	176
F4	1550 ± 40	1.9	1.85 ± 0.14	108	1.75	85	142
F5	990 ± 100	1.6	3.54 ± 0.07	65	1.75	73	116
F1,2,3	3240 ± 70	3.0	1.86 ± 0.05	157	5.00	94	166
OSL-PDR	1580 ± 80	2.0	2.45 ± 0.07	76	1.50	77	108
F1,2,3-PDR	1790 ± 60	2.7	2.27 ± 0.07	85	2.00	83	110

a Nonaggregated.

### Application of Lignin Fractions in PU Coatings

With the aim of highlighting the potential of such tailored lignin fractions for use in industrially relevant applications and to underline the existing correlations between lignin characteristics and material properties, representative PU coatings were prepared by reaction of a hexamethylene-diisocyanate-based polyisocyanate linker (pHMDI from here on) with the as-is lignin (OSL), its (combined) fractions (F1, F4, F5, F1,2,3), or the partially depolymerized lignins (OSL-PDR and F1,2,3-PDR) at different proportions, i.e., at different OH/NCO molar ratios. For this purpose, only the significantly more reactive aliphatic hydroxyl groups in lignin were considered in the stoichiometric calculations, in line with previous studies.^36^

To ensure full dissolution of both lignin and pHMDI under cross-linking reaction conditions (150 °C, 1 h), different solvents were tested (i.e., tetrahydrofuran (THF), methyl ethyl ketone, and ethyl acetate), with THF proving best in completely dissolving the lignin and thus being selected for coating preparation (in line with previous works,^25^ encouraging solubility levels were achieved also using 2-methyl-THF, which can represent a potential biobased alternative in future upscaled applications).

**Figure 5:**
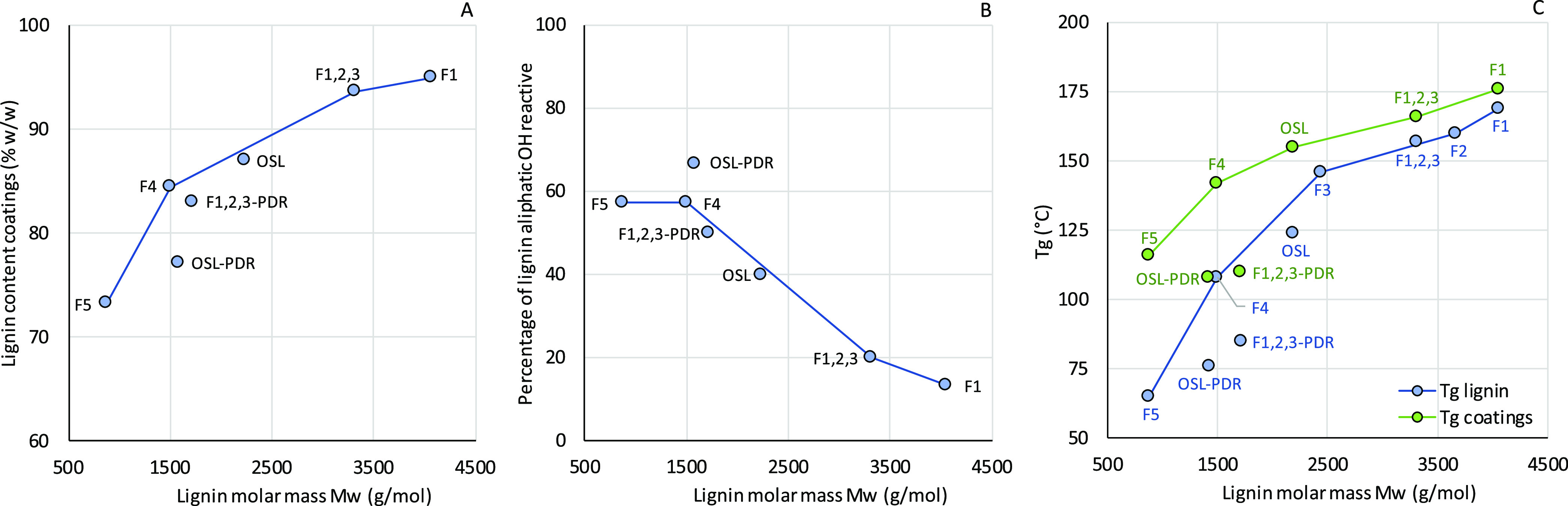
(A) Coating lignin content, (B) percent cross-linked lignin OH groups, and (C) lignin-coating glass transition temperatures.

For each lignin fraction, the optimal composition of the resulting coating was determined on the basis of the following two criteria: on the one hand, the maximum amount of covalently bound lignin incorporated within the coating and a gel content (insoluble mass fraction) yield >90%; on the other hand, the complete conversion of pHMDI NCO groups, as conveniently gauged through monitoring the disappearance of the −NCO stretching signal centered at 2270 cm^–1^ in the Fourier transform infrared (FTIR) spectrum of the resulting coating (see Figure S13 for FTIR spectra). With these criteria, the minimum amount of lignin needed could be defined to enable successful and complete isocyanate conversion (lower OH/NCO ratios would result in the detection of residual unreacted NCO functionalities), thus providing an estimation of the fraction of reactive OH groups in lignin. The obtained optimized OH/NCO molar ratios are shown in Table 1 together with the corresponding lignin mass percentage in the coating with respect to the total dry mass (see also Figure 5A). These coating compositions always allowed for a gel content > 95%, with the only exception of F5 (around 93%). Materials made with a higher lignin content (higher OH/NCO ratio) were excluded from further testing, as more than 10% w/w of free lignin could be extracted from these coatings upon solvent soaking.

For OSL, the optimum aliphatic OH/NCO molar ratio was found to be 2.5, resulting in a coating with 87% lignin content. High-molar-mass lignin fractions (F1, F1,2,3) have a comparable aliphatic OH content as OSL (Table 1), but exhibited lower OH group reactivity toward NCO groups (Figure 5B), most likely due to steric hindrance (i.e., shielding of some OH groups inside the macromolecular structure).^10,11,37^ Indeed, the optimal OH/NCO ratio was shown to increase (from 2.5 for OSL) to 7.5 for F1 and to 5.0 for F1,2,3, with a corresponding increase in lignin contents within the coatings of up to 95 and 94%. Lignin F4, which has a similar amount of aliphatic OH functionalities as OSL but a lower molecular weight, should display a higher OH group reactivity, and the optimal F4-based coating was indeed formulated with a smaller OH/NCO ratio (1.75; 85% lignin content). On the contrary, F5, with twice as many aliphatic OH functionalities as the other lignin fractions and a significantly lower molecular weight, did not show a higher reactivity with pHMDI than the other fractions. Instead, an OH/NCO molar ratio of 1.75 proved optimal for F5 (same as for F4), thus incorporating 73% w/w of lignin in the final material. This result can be explained by considering the high sugar content of F5 (see discussion above), which may prevent the reaction between lignin functionalities and NCO groups. The presence of some unreacted material in this coating was also evidenced by the lower gel content obtained in this case compared with the other systems. Being of relatively low molecular weight, unreacted F5 could in fact be easily extracted with THF from the coating during solvent soaking tests. As mentioned, partial depolymerization of OSL and F1,2,3 reduced the lignin molar mass and dispersity and increased the abundance and reactivity of aliphatic OH groups. As a result, coatings could be made with a lower OH/NCO molar ratio, 1.5 for OSL-PDR and 2.0 for F1,2,3-PDR, i.e., with a higher content of the isocyanate linker (77 and 83% lignin content, respectively). In general, as represented in Figure 5B, the higher the lignin molar mass, the lower the OH reactivity, with the only exception of F5. The increasing OH reactivity from F1 to F4 is also supported by the increasing amount of syringyl units observed in the fractions (Figure 2F), which are known to promote lignin-isocyanate reaction owing to the resonance stabilization effect of the two methoxy moieties during the formation of the intermediate urethane species, as reported in the literature and in our previous works.^11,38^

### Characterization of Lignin-Based PU Coatings

#### Thermal Characterization

The glass transition temperatures (*T*_g_) of the selected lignins as well as the PU coatings were investigated by means of DSC and are shown in Table 1 and Figure 5C. DSC traces are available in Figure S12. Generally, a good correlation is observed between lignin molar mass and *T*_g_, in accordance with similar studies on lignin fractionation.^32,39,40^ In particular, the *T*_g_ goes down when molecular weight goes down from F1 to F5, from a maximum of 169 °C to a minimum of 65 °C, respectively. In view of this, it is worth mentioning that the high excess of lignin used in F1-based PU coatings (i.e., aliphatic OH/NCO = 7.5) can also be related to the low molecular mobility of F1 chains at the cross-linking temperature (150 °C), which made the reaction kinetics slower. As expected, lower *T*_g_ values compared to the parent counterparts are found for the two partially depolymerized lignin samples OSL-PDR and F1,2,3-PDR, which display *T*_g_ values of 76 and 85 °C, respectively (as opposed to *T*_g_ = 124 °C for OSL and 157 °C for F1,2,3).

For all produced coatings, a single glass transition was detected, indicating that a homogeneous PU material is formed. In accordance with the *T*_g_ values registered for the starting lignins, coatings based on OSL, F1, F4, and F1,2,3 exhibit *T*_g_ values of 155, 176, 142, and 166 °C, respectively. Coatings composed of PDR lignins and F5 are characterized by lower *T*_g_, in all cases around 110 °C.

#### Surface Wettability and Characterization

The wettability properties of all of the lignin-based PU coatings were investigated by static contact angle measurements against water and diiodomethane (CH_2_I_2_). The surface tension γ including its dispersive (γ^d^) and polar (γ^p^) components was calculated using the Owens, Wendt, Rabel, and Kaelble (OWRK) method (Table 2).^41^

**Table 2:** Static Contact Angles (θ_H_2_o_, θC_H_2I2__), Total Surface Tension (γ) and Its Dispersive (γ^D^), and Polar (γ^P^) Components for Lignin-Based PU Coatings

	θ_H_2_O_ [deg]	θ_CH_2_I_2__ [deg]	γ^p^ [mN/m]	γ^d^ [mN/m]	γ [mN/m]
PU OSL	84.3 ± 1.0	51.0 ± 2.6	4.7	30.6	35.3
PU F1	76.1 ± 0.9	38.9 ± 2.9	6.7	36.0	42.7
PU F4	71.4 ± 1.7	38.9 ± 2.4	9.1	35.2	44.2
PU F5	77.4 ± 1.0	34.5 ± 1.7	5.5	38.5	44.0
PU F1,2,3	82.7 ± 1.5	40.5 ± 1.2	4.0	36.4	40.4
PU OSL-PDR	75.8 ± 1.5	39.3 ± 0.9	6.8	35.7	42.6
PU F1,2,3-PDR	58.0 ± 3.3	42.9 ± 1.8	16.8	30.8	47.6

All PU materials are slightly hydrophilic. In the literature, the decrease in hydrophobicity of lignin-based PU films is usually correlated with an increase of the lignin content.^11,25,42^ The surface behavior of the materials was found to be affected not only by the lignin content but also by the molecular weight and content of phenolic OH of the considered lignin fraction, as well as by the *T*_g_ of the related PU coating. The highest contact angles (CA) with water (i.e., lower hydrophilicity) were registered for PU systems based on OSL, F1, F1,2,3, and F5, with values of 84.3, 76.1, 82.7, and 77.4°, respectively. In the case of OSL, F1, and F1,2,3, this behavior may be ascribed to the higher molecular weight of the pristine material and to the high *T*_g_ values of these coatings, which prevent the unreacted OH groups in lignin from exposing themselves on the material surface, as observed on other PU coating systems.^43^ In the case of F5-based PU, the high θ_H_2_O_ value may be associated with the low amount of lignin contained in this material (see Table 1). Among all OSL-based fractions, F4-based PU coatings exhibited the lowest water CA (71.4°), likely as a result of its high number of phenolic hydroxyls (see Figure 2E). These can provide increased interactions with the probe liquid, as they are less involved than their aliphatic counterpart in the cross-linking reaction with NCO groups in pHMDI. PU coatings containing PDR lignins show a slightly lower water CA (i.e., higher hydrophilicity) than the parent counterparts. This is attributed to the lower *T*_g_ of these systems (stemming from the lower molecular weight of the corresponding lignin fractions), which may be responsible for enhanced macromolecular mobility at the surface, ultimately providing higher −OH/water interactions.

#### Nanomechanical Characterization

**Figure 6:**
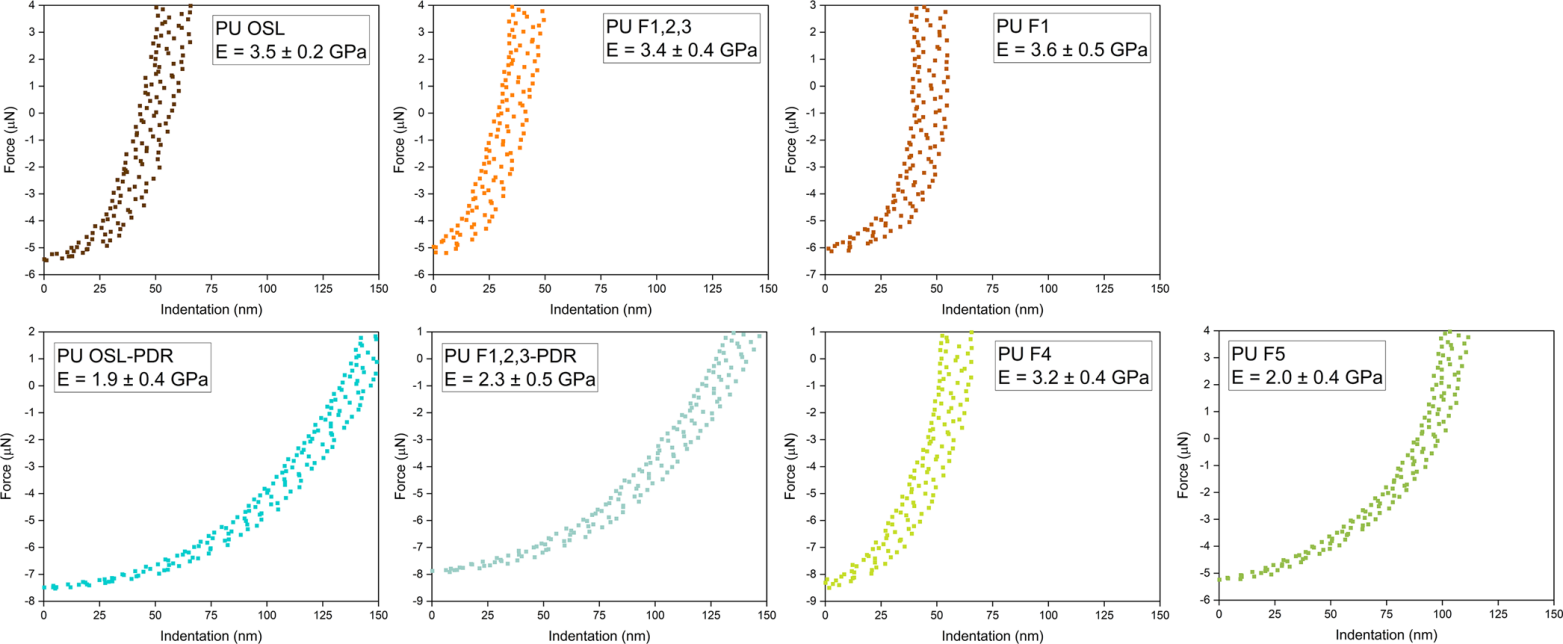
Representative force–distance curves obtained for different lignin-based PU coatings.

Surface mechanical properties of lignin-based PU were studied by means of force–distance curve measurements, performed by atomic force microscopy (AFM). The elastic modulus of each coating was calculated following a procedure described in detail in the Supporting Information. For each sample, four measurements were conducted in different regions of the coating, to allow sufficient reproducibility and statistics. Representative force–distance curves are reported in Figure 6, together with the corresponding extrapolated values of elastic moduli (*E*). Interestingly, an evident correlation between the *T*_g_ of the cross-linked systems and their elastic modulus could be drawn, in line with the literature.^44,45^ In particular, higher values of *E* were recorded for PU materials showing higher *T*_g_, as a result of the relatively lower free-volume fraction, stiffer macromolecular network, and more constrained interchain motion. More specifically, PU systems based on OSL (*T*_g_ = 155 °C), F1 (*T*_g_ = 176 °C), F4 (*T*_g_ = 142 °C), and F1,2,3 (*T*_g_ = 166 °C) fractions were found to exhibit *E* values as high as 3.5, 3.6, 3.2, and 3.4 GPa, respectively. Conversely, PU coatings obtained from F5, OSL-PDR, and F1,2,3-PDR fractions were characterized by slightly lower elastic modulus (i.e., 2.0, 1.9, and 2.3 GPa, respectively), in agreement with their lower *T*_g_ values (i.e., 116, 108, and 110 °C, respectively). Based on these results, OSL elastic modulus was found to be in line with analogous lignin-based PU materials previously reported in the literature.^11,25^ Interestingly, PDR fractions enabled more flexible coatings, thus highlighting the versatility of this approach.

#### Adhesion Measurements

The adhesive strength of all of the prepared lignin-based PU coatings on different substrates (i.e., glass, aluminum, steel, and wood) was assessed by performing pull-off adhesive tests. In Table 3, the adhesive strength of the different coating systems recorded for each substrate is presented. In general, excellent adhesion performance is observed on all substrates irrespective of the type of lignin fraction used, with adhesive strength values in all cases higher than 1 MPa, in line with recent reports.^11,25^ In particular, all PU coatings were found to exhibit high adhesive strength on glass substrates, likely due to the favorable noncovalent interactions between unreacted −OH and polar groups in lignin and the oxygenated moieties on the glass surface. Slightly lower adhesive strengths were instead encountered with coatings on metal substrates (i.e., aluminum and steel), with average higher performance observed on steel as a result of an oxide passivation layer able to better interact with the free hydroxyl moieties in lignin, thus enhancing coating adhesion on this metal. Finally, cohesive detachment of the substrate during the pull-off tests was reported in the case of PU coatings on wood, clearly indicating excellent adhesive interactions. This behavior can be rationalized on the basis of the favorable affinity between lignin and wood, as a result of their common lignocellulosic nature. The favorable adhesive response of these lignin-based PU coatings on different substrates provides additional demonstration of their versatility and potential applicability as protective systems with tailored physicochemical properties.

**Table 3:** Adhesive Strength of Lignin-PU Coatings on Different Substrates

	adhesion strength [MPa]			
	glass	wood	aluminum	steel
PU OSL	8.8 ± 2.4	>9.0^a^	1.3 ± 0.2	1.8 ± 0.4
PU F1	5.8 ± 2.2	>9.0^a^	1.3 ± 0.3	1.2 ± 0.2
PU F4	7.8 ± 1.7	>9.0^a^	1.7 ± 0.3	2.1 ± 0.4
PU F5	6.0 ± 0.8	>9.0^a^	1.6 ± 0.2	4.5 ± 1.5
PU F1,2,3	6.3 ± 0.7	>9.0^a^	1.8 ± 0.3	2.0 ± 0.3
PU OSL-PDR	5.5 ± 0.1	>9.0^a^	2.0 ± 0.3	2.0 ± 0.2
PU F1,2,3-PDR	4.2 ± 2.0	>9.0^a^	1.3 ± 0.2	1.2 ± 0.2

a Readout limit of the pull-off test instrument.

## Conclusions

Fractionation of beech wood organosolv lignin proved to be an effective strategy for producing lignin fractions with distinctly different characteristics and lower polydispersity. As expected, high molar mass fractions generally show a higher abundance of lignin interunit linkages, a lower phenolic hydroxyl group content, and a lower S/G ratio as compared to the lower molar mass fractions. Reduction of lignin molar mass and dispersity by reductive partial depolymerization was especially effective for the larger lignin fragments having a higher β-O-4 content. The PDR process was successfully scaled-up to a 50 L reactor using OSL and combined high-molar-mass fractions, enabling larger-scale production of PDR lignin.

High-quality lignin-based PU coatings were produced with OSL, certain lignin fractions, and the PDR lignins. The use of lignin fractions with different properties (i.e., molecular weight, OH functionalities, functional group reactivity) provided access to coatings with tunable properties. In particular, high-molecular-weight lignins gave coatings that had a high *T*_g_ and were more rigid and hydrophobic. In contrast, low-molecular-weight lignins led to PU coatings with lower *T*_g_, more hydrophilic behavior, and enhanced flexibility.

In general, it was demonstrated that the effect of the PDR process is to increase the lignin reactivity toward the NCO groups, as a result of the reduction in the lignin molecular weight. Coatings produced from PDR lignin showed the lowest *T*_g_ values and improved coating flexibility, showing additional effect of lignin structural modification during the depolymerization process.

Overall, selective lignin fractionation and partial depolymerization proves to be a versatile strategy for the development of coating applications with tailored properties and significantly lower cross-linker content than the typical fossil-based ones. The established relationships between the lignin structure and PU coating performance provide support to further develop fully biobased and isocyanate-free PU materials with maximized sustainability impact. To this extent, follow up research focuses on the use of affordable, safe, and sustainable biobased cross-linkers (available at the kilogram scale). Indeed, the limited residual ether content of PDR lignins is anticipated to be a valuable asset for the development of resilient polyols for such circular coating formulations.

## Supporting Information Available

Additional experimental details, materials, methods, and 2D-HSQC NMR spectra (PDF)
