# Supplementary material for: Tuning the Properties of Biobased PU Coatings via Selective Lignin Fractionation and Partial Depolymerization
Source: ACS Sustain Chem Eng. 2023 Apr 21;11(18):7193–202. doi: 10.1021/acssuschemeng.3c00889 (PMC10171370; doi:10.1021/acssuschemeng.3c00889)
Supplement: Supplementary file 1 — sc3c00889_si_001.pdf [file sc3c00889_si_001.pdf]

Supporting Information

Tuning the Properties of Biobased PU Coatings via Selective Lignin Fractionation and Partial Depolymerization.

Arjan T. Smit,[a,c] Emanuela Bellinetto,[b] Thomas Dezaire,[c] Oussama Boumezgane,[b] Luke A. Riddell,[c] Stefano Turri,[b] Michiel Hoek, [a] Pieter C. A. Bruijnincx [c] and Gianmarco Griffini*[b]*

a. The Netherlands Organisation for Applied Scientific Research (TNO), unit Energy Transition, Biobased & Circular Technologies group. P.O. Box 1, 1755 ZG Petten, The Netherlands.

b. Department of Chemistry, Materials and Chemical Engineering "Giulio Natta", Politecnico di Milano, Piazza Leonardo da Vinci 32, 20133 Milano, Italy

c. Organic Chemistry and Catalysis, Institute for Sustainable and Circular Chemistry, Utrecht University, Universiteitsweg 99, 3584 CG Utrecht, The Netherlands.

* Email:

arjan.smit@tno.nl

gianmarco.griffini@polimi.it

Number of pages: 20

Number of figures: 13

Number of tables: 4

Number of schemes: 0

Contents

Lignin fractionation 4
Lignin Partial Depolymerization by Reduction (PDR) 11
Application of lignin in coatings 17

List of Tables

Table S1. Assignments and correction factors for integral regions of HSQC NMR spectra 5
Table S2. Characteristics of OSL lignin and its fractions. 8
Table S3. Characteristics of depolymerized OSL fractions. 16
Table S4. Characteristics of (larger scale depolymerized) OSL and mixture of high molar mass fractions. 16

List of Figures

Figure S1. Yield of lignin fractions using step-wise liquor dilution with water. 7
Figure S2. Reproducibility of the OSL lignin fractionation experiments and Size Exclusion Chromatography curves of the OSL fractions. 7
Figure S3. Aromatic/unsaturated region of the 2D-HSQC NMR spectra of OSL and OSL fractions. 9
Figure S4. Oxygenated aliphatic region of the 2D-HSQC NMR spectra of OSL and OSL fractions.. 10
Figure S5. Process conditions for partial depolymerization of OSL. 11
Figure S6. Process conditions for partial depolymerization of F1,2,3. 12
Figure S7. SEC curves of isolated, non-aggregated (NA-) and partially depolymerized OSL and its fractions. 13
Figure S8. SEC curves of isolated, non-aggregated (NA-) and partially depolymerized OSL and its fractions using a logarithmic x-axis scale. 14
Figure S9. SEC curves of larger scale depolymerized OSL. 15
Figure S10. Molar mass distribution of isolated, non-aggregated (-NA) and partially depolymerized OSL fractions. 15
Figure S11. Aliphatic and total phenolic hydroxyl content of (depolymerized) OSL and its fractions. 16
Figure S12. (A) DSC curves of pristine lignin (OSL), lignin fractions (F1-F5), depolymerized OSL (OSL-PDR) and depolymerized fraction mixture (F1,2,3-PDR); (B) DSC curves of the corresponding PU coatings. 19
Figure S13. FTIR spectra of all lignin-based PU coating formulations before curing (pre-curing) and after curing (post-curing). 20

Lignin fractionation

Experimental

For lignin fractionation, 25 g of lignin (oven dry weight) was dissolved overnight in 250 mL of 60 % w/w aqueous acetone (corrected for lignin moisture content) as described in previous work.[1] Afterwards, the sample was centrifuged at 3488 *g* for 5 min. The liquid was decanted into a new pre-weighed 750 mL centrifuge pot and demineralized water was added to obtain a 55% w/w aqueous acetone solution which was placed in an IKA shaking incubator (100 rpm) at room temperature for 30 min. The liquid was again centrifuged and decanted. This procedure was repeated to obtain lignin precipitate from (diluted) liquors containing 60% to 10% w/w aqueous acetone. All lignin pellets were dried at 60 °C overnight and weighed. The remaining liquid was dried completely in a rotary evaporator at 60 °C and represents the fraction which was soluble in less than 10% w/w aqueous acetone. The procedure was conducted as single experiments on Alcell lignin, beech wood lignin obtained from ethanol organosolv fractionation (HT EtOH OS) and beech wood lignin obtained from pilot-scale acetone organosolv fractionation (P-BEC-3, OSL in this study). The beech wood lignin characteristics are described in previous work.[1]

For larger scale lignin fractionation three identical experiments were conducted. For each experiment 500 g of OSL lignin (dry weight) was dissolved overnight at room temperature in 5 L of 60% w/w aqueous acetone. Afterwards, the sample was centrifuged at 3488 *g* for 5 min. The liquid was decanted into a new 10 L beaker and demineralized water was added to obtain a 45% w/w aqueous acetone solution which was placed at room temperature for 30 min. The liquid was again centrifuged and decanted. This procedure was repeated to obtain lignin precipitate from (diluted) liquors containing 40% and 30% aqueous acetone. To obtain lignin precipitate at a 15% acetone concentration, the lignin solution in 30% acetone was placed in a Buchi 20 L rotary evaporator where acetone was removed at 60 °C and 200 mbar pressure. The acetone free liquid (approx. 6 kg) was decanted in a 10 L beaker. The lignin attached to the wall of the 20 L rotary evaporator roundbottom flask was dissolved using condensate (approx. 1 kg). The liquid was then mixed with the decanted liquid. The amount of used condensate was calculated to obtain a 15% w/w acetone solution after mixing with the decanted liquid (assuming a condensate acetone concentration of 100%). The liquid was then cooled and centrifuged to obtain the precipitated lignin. The supernatant was transferred to the 20 L rotary evaporator to remove water and solvent at 60 °C and obtain the remaining lignin as a dry powder. All other lignin pellets were dried in the 750 mL centrifuge pot in a conventional oven at 60 °C overnight and weighed. The obtained lignin fractions from the triplicate experiments were combined per fraction, milled and sieved over 250 µm.

Lignin sugar content was determined using modified versions of the NREL standard biomass analytical procedures (NREL/TP-510-42618).[1-4] In short, the content of lignin and carbohydrates was determined in duplicate as follows: the sample was hydrolyzed in two steps: (1) 12 M (72% w/w) H_2SO_4 (30 °C, 1 h) and (2) 1.2 M H_2SO_4 (100 °C, 3 h). The solid residue was determined gravimetrically and its ash content was measured. The acid-insoluble lignin (AIL) content was based on the amount of ash-free residue, and acid-soluble lignin (ASL) was determined using UV–VIS absorption. Finally, the hydrolysate was analyzed for monomeric sugars and corrected for sugar degradation. Biomass ash content was determined according to NREL/TP-510-42622.[5]

Analysis of monomeric sugars was performed as described in previous work[4] by HPAEC-PAD (ICS3000, Dionex) equipped with a CarboPac PA1 column and a post column addition of 0.2 mL/min 0.25 M NaOH. A gradient of NaOH was used as eluent (0.25 mL/min): 15 mM (0–1 min), 0 mM (1–21 min), increasing from 0 to 187.5 mM (21–37 min), 250 mM (37–42 min), decreasing from 250 to 15 mM (42.0-42.1 min) and 15 mM (42.1-50 min). Lactose was used as an internal standard. Samples containing 1.2 M sulfuric acid from biochemical composition analysis were neutralized with barium carbonate and centrifuged before analysis.

Alkaline Size Exclusion Chromatography (SEC) was conducted in triplicate analysis as described in previous work[4] using a Thermo ICS-3000 system equipped with a thermostatic column oven operating at 40 °C and a Thermo VWD detector. 50 mg lignin was solubilized in 50 mL 0.5 M NaOH. Injection volume was 100 µL. A commercially available column packed with PSS MCX, 5 µm particle size, 500A porosity, designed for polymers with a molecular size between 100-35.000 Da was used. The eluent was a solution of 0.5 M NaOH with a flow rate of 1 mL/min. An ultraviolet detector was used at 280 nm. The column was calibrated with poly(styrene sulfonate) sodium salts of different molecular sizes (94, 1830, 3610, 4900, 10.600, 13.200, 16.800, 29.100 and 32.900 Da).

High-field 2D NMR spectra were obtained as described in previous work[4] on a Bruker Ultra 600 MHz spectrometer equipped with a Bruker 5 mm inverse triple resonance probe as reported in earlier work.[1] ^{1}H-^{13}C Heteronuclear single quantum coherence spectroscopy (HSQC) spectra were recorded using the pulse sequence hsqcetgpsp.3 and the following parameters: A spectral window of 13 to -1 ppm was applied in F2 (1H) with 2048 collected datapoints and in F1 (13C), a

spectral window of 160 to 0 ppm was applied with 256 scans, an interscan delay of 1 s and cnst2 set to 145 Hz. The spectra were processed with MestreNova software by first automatic baseline correction (polynomial fit 3rd order), followed by automatic phase correction. Apodisation was then performed by application of Gaussian (GB = 0.1), exponential functions (LB = 0.3 Hz) and a squared sine-bell function at 90° in F2, and a squared sine-bell function at 90° in F1.

NMR samples were prepared by stirring approximately 200 mg of lignin in 750 µL DMSO-d6 overnight to ensure total dissolution. The chemicals shifts were referenced using the solvent signal as the shift reference (δC 39.6, δH 2.49 ppm). Semi-quantitative analysis of linkages was performed by using a section of the aromatic region as an internal standard, which was performed by setting the $S_{2,6}/2$ and G_2 integrals to 100 (i.e. the S region contains two C-H correlations so needs to be halved). The $S_{2,6}$ integral included the $S_{2,6}$, $S'_{2,6}$, $SA_{2,6}$, $AS_{2,6}$, $X2S'_{2,6}$ cross peaks and the G_2 integral the G_2, G'_2, AV_2, VA_2, V_2 and $X2G'_2$ cross peaks. The G_2 integral was further corrected for F_4 by subtracting the better isolated F_5 signal. The unknown peak at δC/δH 112.6/6.6 was cut from the G_2 integral directly.
The alkyl area integral included DMSO-d6 and acetone peaks and were corrected accordingly. Peaks of interest were then referenced to the aforementioned aromatic region which then allowed for the expression of linkage integral values per 100 aromatic units (/100 Ar) as shown in Equation 1 where X denotes the unit of interest.

$$X = \frac{\int X}{\int \frac{S_{2,6}}{2} + \int G_2} \times 100$$

$$Equation\ 1.$$

Some the chosen integral regions of linkages correspond to multiple C-H correlations so correction factors must be applied as shown in Table S1 below.

Table S1. Assignments and correction factors for integral regions of HSQC NMR spectra

Notation	Chemical Shift (δ / ppm)	Assignment	Factor
$-OCH_3$	55.6/3.73	C–H in methoxyls	1
$S_{2,6}$	104.5/6.76	$H_{2,6}-C_{2,6}$ in syringyl units	0.5
$S'_{2,6}$	106.3/7.38	$H_{2,6}-C_{2,6}$ in oxidized (Cα = O) syringyl units	0.5
$SA_{2,6}$	107.1/7.21	$H_{2,6}-C_{2,6}$ in syringaldehyde units	0.5
$AS_{2,6}$	104.1/7.13	$H_{2,6}-C_{2,6}$ in acetosyringone units	0.5
$X2S'_{2,6}$	106.2/7.06	$H_{2,6}-C_{2,6}$ in sinapaldehyde units	0.5
G_2	111.1/7.05	C_2-H_2 in guaiacyl units	1
G'_2	112.1/7.56	C_2-H_2 in oxidized (Cα = O) guaiacyl units	1
V_2	110.3/7.38	C_2-H_2 in vanillin units	1
V_6	126.0/7.43	C_6-H_6 in vanillin units	1
VA_2	109.9/7.48	C_2-H_2 in vanillic acid units	1
VA_6	123.3/7.46	C_6-H_6 in vanillic acid units	1
AV_2	112.3/7.46	C_2-H_2 in acetovanillone units	1
AV_6	123.2/7.51	C_6-H_6 in acetovanillone units	1
$X2G'_2$	110.9/7.32	C_2-H_2 in coniferaldehyde units	1
$X2G'_6$	118.9/7.32	C_6-H_6 in coniferaldehyde units	1
A_α	71.5/4.82	$C_\alpha-H_\alpha$ in β-O-4′ substructures	1
A_β	83.6/4.4 (G) and 86.1/4.2 (S)	$C_\beta-H_\beta$ in β-O-4′ substructures	1
A_γ	59.8/3.5	$C_\gamma-H_\gamma$ in γ– hydroxylated β-O-4′ substructures	1
A'_β	83.0/5.28	$C_\beta-H_\beta$ in α-oxidized β-O-4′ substructures	1

B_α	87.3/5.48	C_α–H_α in phenylcoumaran substructures	1
B_β	53.1/3.50	C_β–H_β in phenylcoumaran substructures	1
B_γ	62.6/3.74	C_γ–H_γ in phenylcoumaran substructures	1
C_α	85.2/4.69	C_α–H_α in β–β′ resinol substructures	0.5
C_β	53.7/3.08	C_β–H_β in β–β′ resinol substructures	0.5
C_γ	71.2/4.2 and 71.3/3.8	C_γ–H_γ in β–β′ resinol substructures	0.5
C'_α	86.9/4.35	C_α–H_α in β–β′ epiresinol substructures	1
$C'_{\alpha'}$	81.4/4.76	$C_{\alpha'}$–$H_{\alpha'}$ in β–β′ epiresinol substructures	1
$C'_{\beta'}$	68.5/3.1 and 68.5/3.8	$C_{\beta'}$–$H_{\beta'}$ in β–β′ epiresinol substructures	1
C'_γ	53.9/2.83	C_γ–H_γ in β–β′ epiresinol substructures	1
J_α	153.9/7.59	C_α–H_α in cinnamaldehyde end-groups	1
J_β	126.1/6.81	C_β–H_β in cinnamaldehyde end-groups	1
$SB1_\alpha$	125.6/6.96	C_α–H_α in *trans*-stilbene substructures (β-1)	0.5
$SB2_\beta$	119.8/7.24	C_β–H_β in *trans*-stilbene substructures (β-5)	1
Hk_α	44.6/3.62	C_α–H_α in Hibbert ketone structures	1
Hk_γ	67.2/4.20	C_γ–H_γ in Hibbert ketone structures	1
F_3	122.8/7.53	C_3– H_3 in furfural	1
F_4	112.8/6.76	C_4– H_4 in furfural	1
F_5	149.1/8.09	C_5– H_5 in furfural	1
HMF_3	124.1/7.48	C_3– H_3 in 5-hydroxymethylfurfural	1
HMF_4	109.6/6.61	C_4– H_4 in 5-hydroxymethylfurfural	1
HMF_6	56.0/4.53	C_6– H_6 in 5-hydroxymethylfurfural	0.5

^{31}P NMR measurements were conducted using a standard phosphitylation procedure on a Varian 400 MHz NMR spectrometer. A solvent mixture composed of pyridine/deuterated chloroform (1.6/1.0 %v/v) was protected from moisture with 3 Å molecular sieves. Stock solutions of the internal standard (cyclohexanol, 19 mg/mL) and the relaxation agent (chromium(III)acetylacetonate, 11.4 mg/mL) were prepared separately by dissolution in the pyridine/deuterated chloroform solvent mixture. Before dissolution, lignin samples were dried at 60 °C in a sand bath at reduced pressure on a Schlenk line. 40 mg of the dried lignin was dissolved in 500 µL of the solvent mixture by stirring overnight at room temperature. Approximately 1 hour before analysis, 200 µL of the internal standard stock solution and 50 µL of the relaxation agent stock solution were added to the lignin solution. Approximately 30 minutes before analysis, 100 µL of the phosphitylation agent (2-chloro-4,4,5,5-tetramethyl-1,3,2-dioxaphospholane) was added to the sample under continuous stirring. After approximately 5 minutes, the mixture was transferred into a 5-mm-OD NMR tube. ^{31}P NMR spectra were acquired on a Varian 400 MHz NMR spectrometer using a standard phosphorus pulse programme with a relaxation delay of 10 s and 256 or 512 acquired scans. Chemical shifts were referenced from the sharp signal arising from the reaction product between residual water and 2-chloro-4,4,5,5-tetramethyl-1,3,2-dioxaphospholane at 132.2 ppm.

Results

Lignin fractionation using a dilutive approach with water creates specific fractions with different molar mass distributions and a lower dispersity. Differences in lignin characteristics can be observed in the obtained lignin fractionation yield profile. Figure S1 shows the fractionation yields of Alcell lignin, high temperature ethanol organosolv lignin (190 °C, 60 min, 5 L 60% w/w aqueous ethanol/kg dry weight beech, 10 mM sulfuric acid) and the acetone organosolv lignin (OSL) used in this study. Contrary to OSL, both Alcell and Beech 190 °C Ethanol lignin are partly insoluble in 60% w/w acetone. Most Alcell lignin is precipitated and collected in the fraction with 50% acetone and diminishing yields are obtained in the fractions thereafter. Beech 190 °C Ethanol lignin shows a high precipitation yield in the 45% w/w acetone fraction. OSL lignin mostly precipitates at lower acetone concentrations between 35-45% w/w acetone and shows highest precipitation yields at lower acetone concentrations (<35% w/w acetone) as compared to the other lignins. The precipitation "fingerprint" is likely the result of the combined effects of lignin molar mass and hydrophobicity.
Larger-scale fractionation experiments to produce sufficient lignin quantity for depolymerization and application in polyurethane coatings show a good reproducibility regarding lignin yield in each fraction as shown in Figure S2.

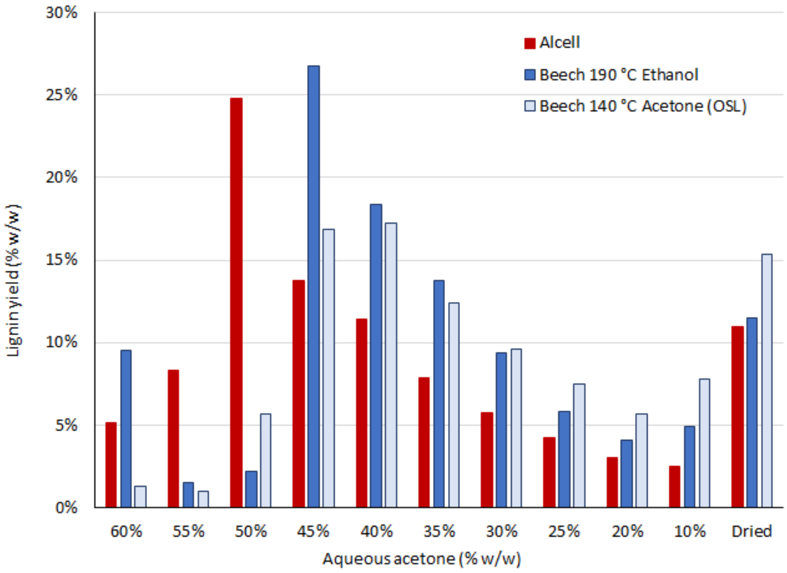

Figure S1. Yield of lignin fractions using step-wise liquor dilution with water.

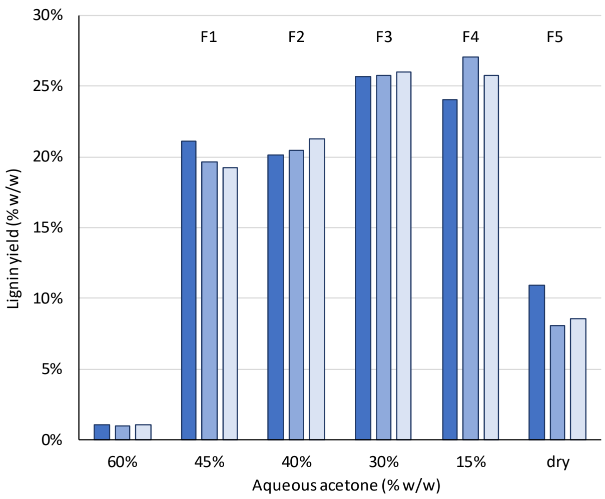

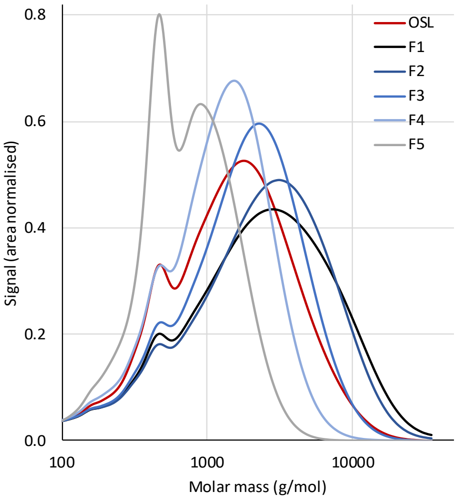

Figure S2. Reproducibility of the OSL lignin fractionation experiments (left), Size Exclusion Chromatography curves of the OSL fractions (right).

Table S2. Characteristics of OSL lignin and its fractions.

			OSL	F1	F2	F3	F4	F5	SWF[a]
Fractionation yield									
	Run 1		100	21.2	20.1	25.7	24.0	10.9	
	Run 2			19.6	20.5	25.8	27.1	8.1	
	Run 3			19.3	21.3	26.0	25.8	8.5	
	Lignin sugar content (% w/w)		1.0	0.4	0.4	0.3	0.6	7.7	1.1
Molar mass[b]									
	Untreated	M_n	1060	1430	1260	1100	890	650	
		M_w	2830	5930	3670	2470	1610	990	3090
		M_w/M_n	2.7	4.2	2.9	2.3	1.8	1.5	
	Non-agglomerated	M_n	950	1130	1160	1010	810	620	
		M_w	2230	3940	3600	2420	1550	990	2640
		M_w/M_n	2.4	3.5	3.2	2.4	1.9	1.6	
Hydroxyl groups content[c]									
	Aliphatic OH		1.89	2.01	1.86	1.75	1.85	3.54	2.03
	5-substituted OH		2.40	2.00	2.13	2.43	2.74	2.62	
	G-OH		0.77	0.70	0.72	0.80	0.84	0.89	
	p-hydroxyphenyl OH		0.09	0.08	0.09	0.10	0.09	0.13	
	Total PhOH		3.25	2.78	2.94	3.33	3.67	3.63	3.30
	Free COOH		0.01	0.03	0.04	0.03	0.04	0.05	
	COOH		0.15	0.14	0.15	0.17	0.21	0.36	0.19
Quantification of identified 2D-HSQC NMR structures									
Aromatic units[d]	Guaiacyl	(G_2)	37.9	40.7	39.8	37.7	33.9	31.3	37.7
	Syringyl	($S_{2/6}$)	62.1	59.3	60.2	62.3	66.1	68.7	63.6
	p-Hydroxyphenyl	($H_{2/6}$)	0	0	0	0	0	0	0
Linkages[e]	β-Aryl ether	(A_α)	8.2	12.5	10.7	10.1	7.4	5.1	9.7
	Oxidised β-Aryl ether	(A'_α)	1.8	1.9	2.1	2.0	1.9	1.3	
	Phenylcoumaran	(B_α)	2.0	2.4	2.9	2.6	1.8	1.4	2.3
	Resinol	(C_α)	3.8	4.7	4.4	3.7	3.5	3.9	4.1
	Epiresinol	(C'_α)	2.1	4.2	3.9	3.4	3.2	3.5	3.6
Other[e]	Hibbert's ketones	(Hk_γ)	4.7	4.2	3.9	3.8	3.9	5.5	4.1
	Cinnamaldehyde	(J_β)	0.8	0.6	0.8	0.1	0.3	0.9	
	Stilbene	($SB1_\alpha$)	0.2	0.2	0.2	0.1	0.2	0.0	
		($SB2_\beta$)	0.2	0.3	0.3	0.3	0.3	0.2	
	Furfural	(F_3)	1.7	0.8	0.8	0.6	0.7	1.7	0.8
	HMF	(HMF_4)	0.6	0.6	0.7	0.4	0.5	1.2	0.6

[a] SWF: Sum Weighed Fractions. [b] Determined by Size Exclusion Chromatography of untreated and non-aggregated lignin, values in g/mol. [c] Determined by ^{31}P NMR, mmol OH groups/g lignin. [d] Aromatic units expressed as percent of S + G. [e] Linkages expressed per 100 aromatic units (S + G).

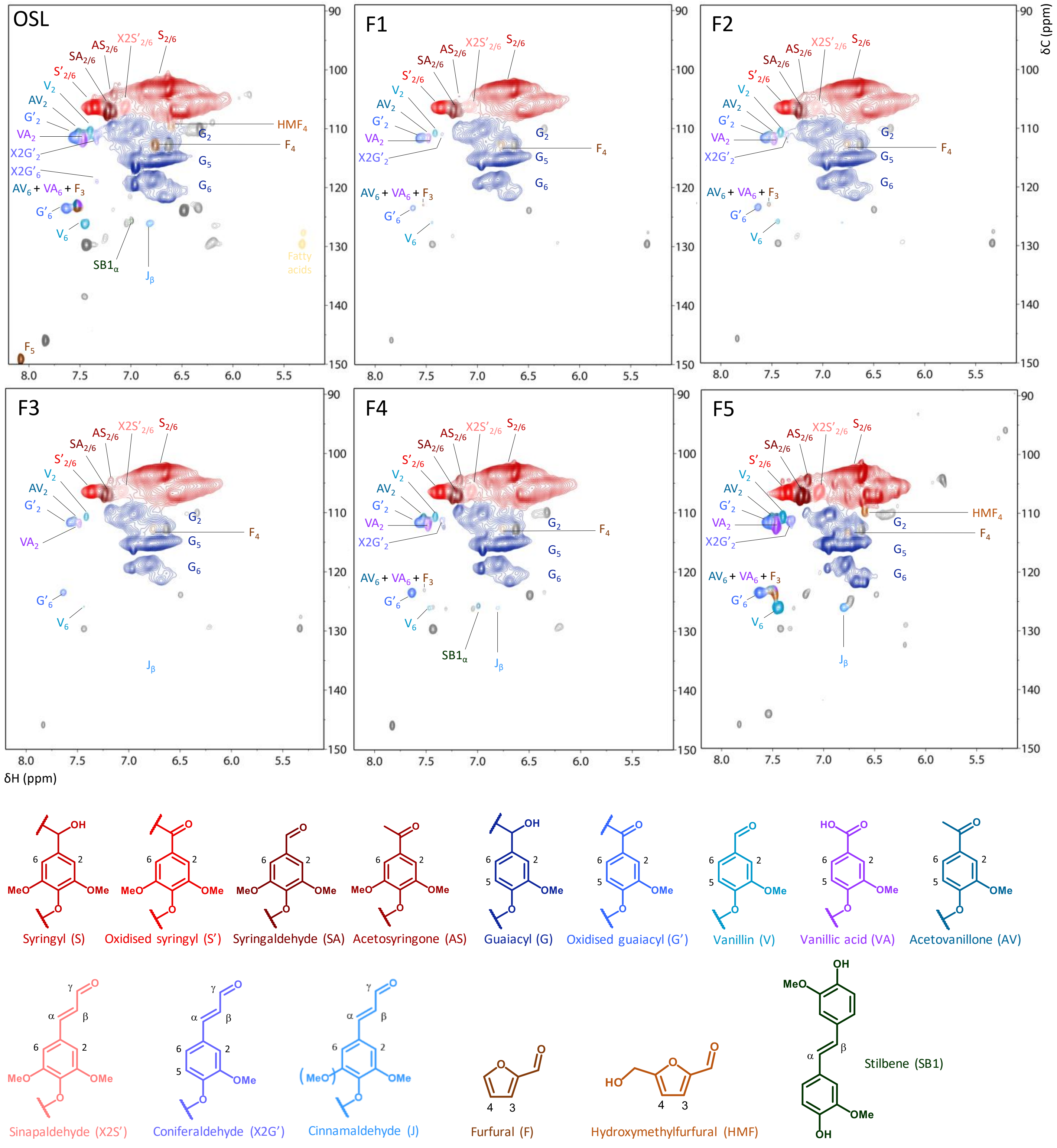

Figure S3. Aromatic/unsaturated region of the 2D-HSQC NMR spectra of OSL and OSL fractions. The main identified structures are shown at the bottom.

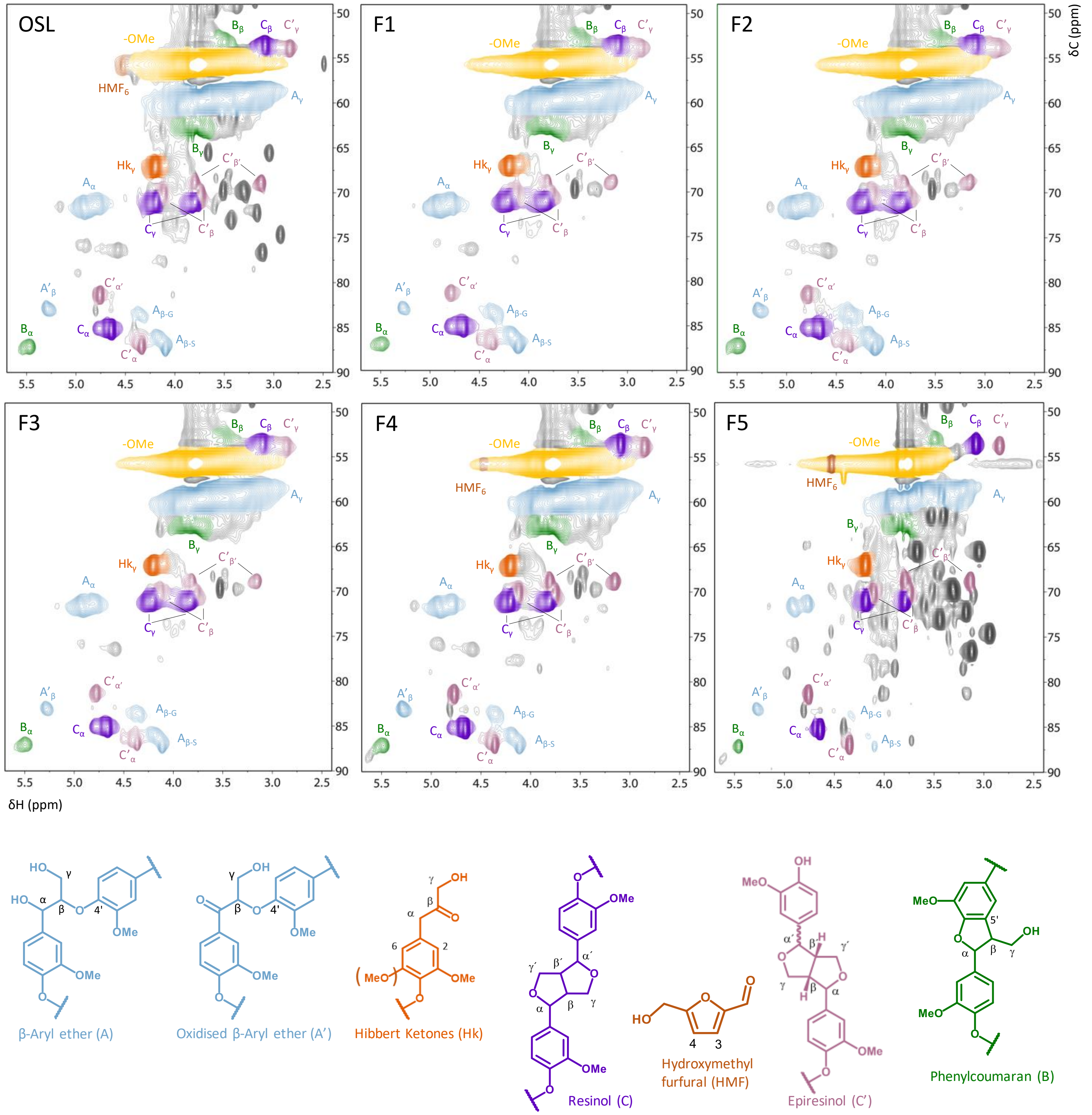

Figure S4. Oxygenated aliphatic region of the 2D-HSQC NMR spectra of OSL and OSL fractions. The main identified structures are shown at the bottom.

Lignin Partial Depolymerization by Reduction (PDR)

Experimental

Lab-scale lignin partial depolymerization was conducted in single experiments using OSL and the (mixed) lignin fractions as recently reported.[6] A 100 mL autoclave containing a mixture of 60 mL methanol, 0.6 g lignin and 0.3 g catalyst (5% w/w Ru/C) was flushed three times with argon and pressurized at room temperature to 30 bar with hydrogen. The mixture was heated to 200 ^oC and kept isothermal for 2 h while stirring at 750 rpm. The depolymerized lignin was isolated from the mixture using previously described procedures.

Larger scale PDR was conducted by Fraunhofer CBP (Leuna, Germany) in a 50 L stirred autoclave.
Partial depolymerization of OSL: 30 L of methanol was transferred to the stirred tank reactor. 900 g OSL lignin and 450 g of 5% Ru/C were then added under stirring. After flushing the reactor three times with nitrogen, a hydrogen pressure of 20 bar was set. The reactor was heated to 225 °C. Up to a temperature of 210 °C, the methanol expanded to 45 L, which resulted in an emergency shutdown due to triggering a fill level alarm. Overall, the temperature inside the reactor remained above 200 °C for 45 minutes. The decrease of the pressure as a result of the reaction was approx. 9 bar (20.1 to 11.2 bar). After cooling, the reactor was emptied using an immersion tube. The catalyst was separated from the reaction solution by pressure filtration at 5 bar through a candle filter. Afterwards, the reactor and the catalyst were washed with 22.5 L of an 80 % aqueous acetone solution. After combination of the washing solution with the reaction liquid, the solvents were removed by distillation at 40 °C with a stirring speed of 200 rpm with a pressure of 260 mbar. A volume of 7.5 L was obtained. The 7.5 L were further concentrated in a rotary evaporator to 1 L. The 1 L partially cleaved lignin solution was further concentratedand dried in an evaporation dish in an oven at 50 °C.

Partial depolymerization of F1,2,3: The combined fractions F1, F2 and F3 were obtained by two additional lignin fractionation series using 2 kg of OSL showing a similar fraction yield distribution as shown in Figure S1 (i.e., 18.9%, 18.4%, 25.1%, 22.0% and 13.0% for F1, F2, F3, F4 and F5, respectively). To avoid another emergency shutdown, the volume was reduced from 30 L to 25 L of methanol and the temperature reduced to 200 °C. For the partial depolymerization, 25 L of methanol was transferred to the stirred tank reactor. 750 g lignin (227 g F1, 221,5 g F2, 301,5 g F3) and 375 g of the catalyst Ru/C were then added under stirring. After flushing the reactor three times with nitrogen, a hydrogen pressure of 20 bar was set. The reactor was heated to 200 °C. After 120 min the reactor was cooled down. The decrease of the pressure as a result of the reaction was approx. 5 bar (20.5 to 15.5 bar). After cooling, the downstream processing was followed as described above.

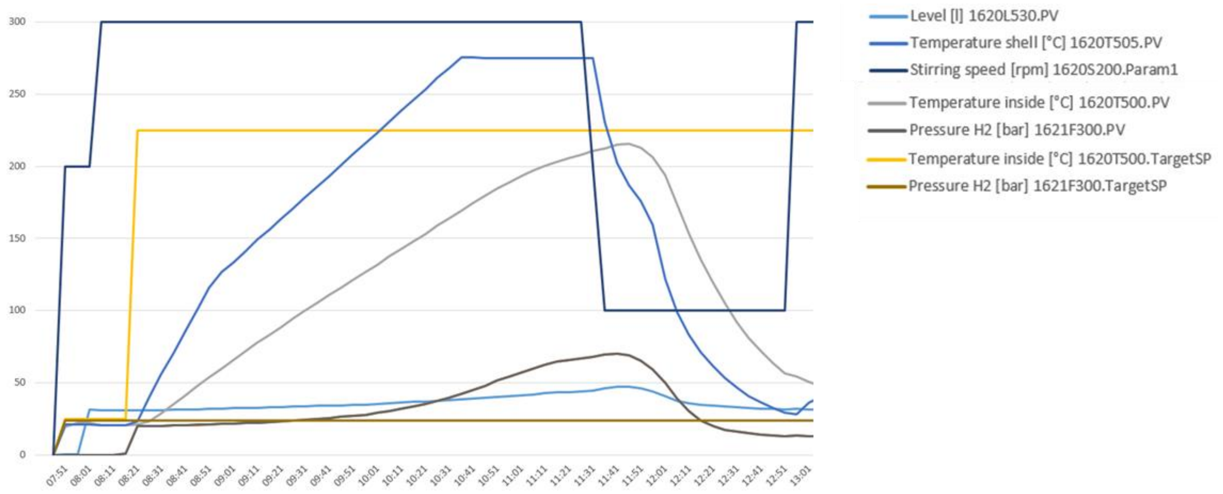

Figure S5. Process conditions for partial depolymerization of OSL.

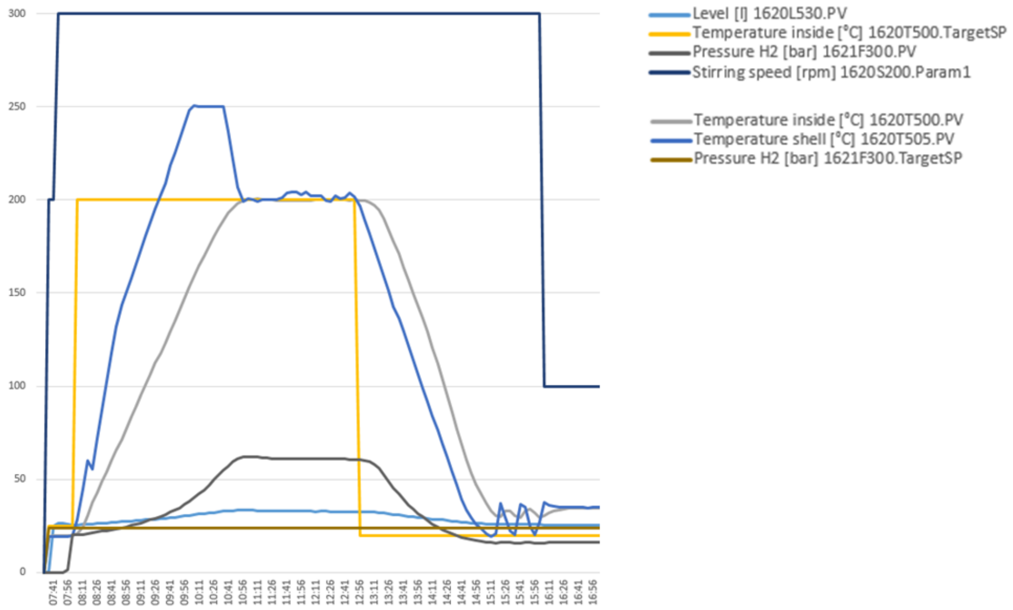

Figure S6. Process conditions for partial depolymerization of F1,2,3.

Results

Figure S7 and Figure S8 show the SEC curves of OSL and its fractions and the curves after de-aggregation (-NA) and partial depolymerization (PDR) using a linear and a logarithmic scale, respectively. Lignin aggregates are only observed in OSL and the F1 fraction. Figure S9 shows the SEC curves of depolymerized OSL, analyzed in separate SEC runs over a period of approximately one year. The variation in M_w shows how the SEC method is sensitive to slight variations in the measurement and obtained curves. Figure S10 provides a breakdown of the SEC analysis in defined molar mass ranges, providing additional insight in the molar mass distribution of lignin.

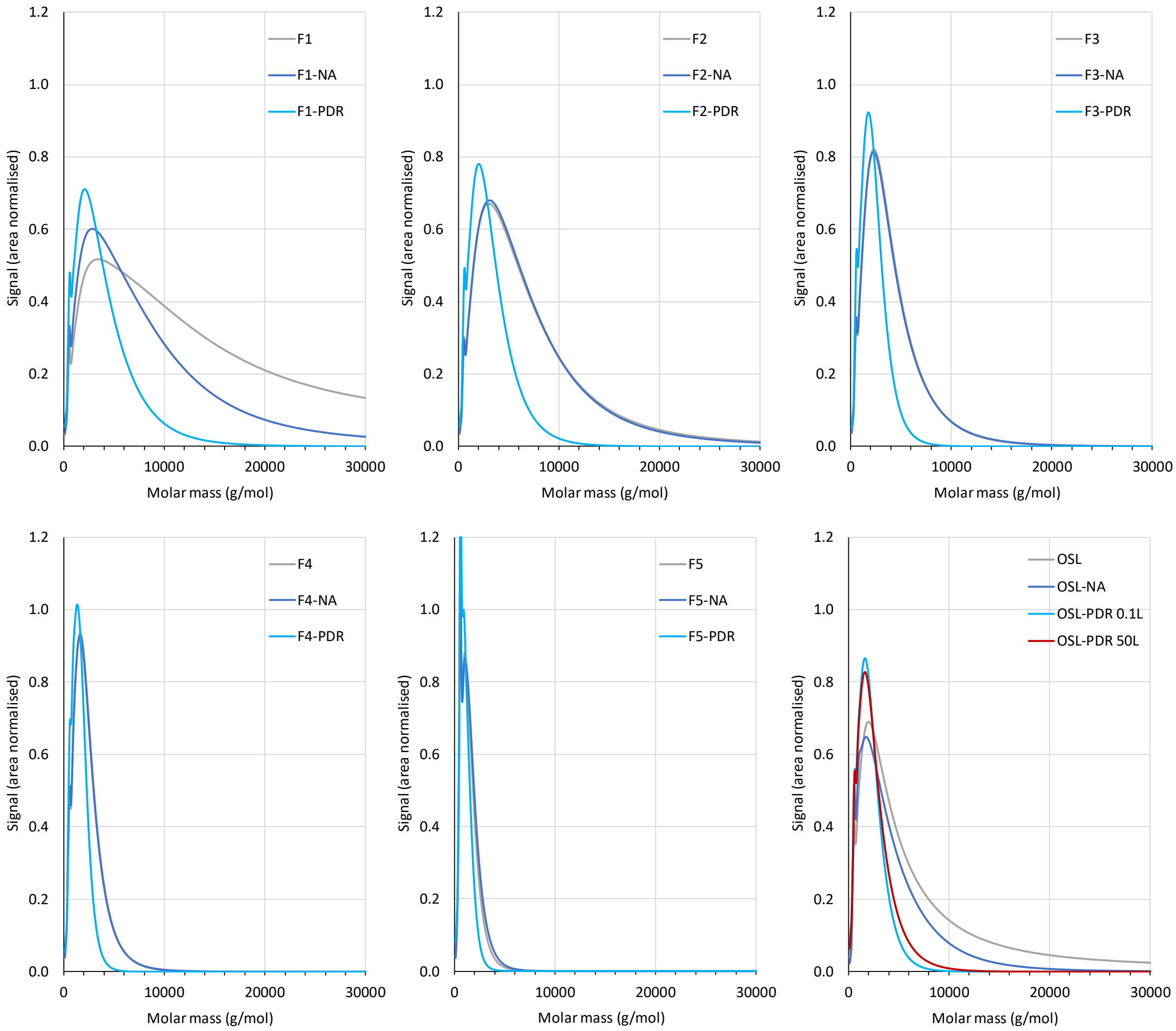

Figure S7. SEC curves of isolated, non-aggregated (NA-) and partially depolymerized OSL and its fractions.

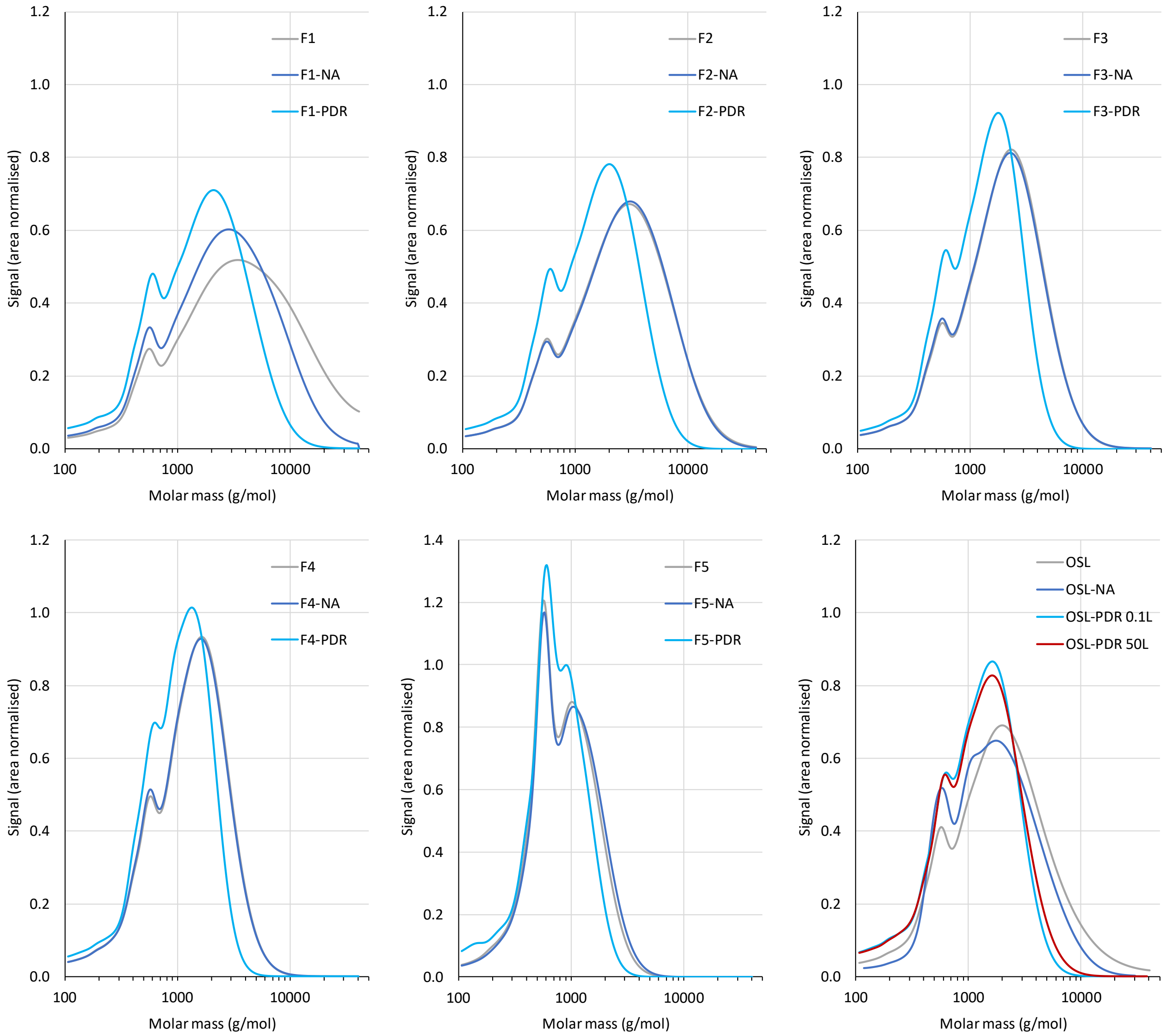

Figure S8. SEC curves of isolated, non-aggregated (NA-) and partially depolymerized OSL and its fractions (Figure S7) using a logarithmic x-axis scale.

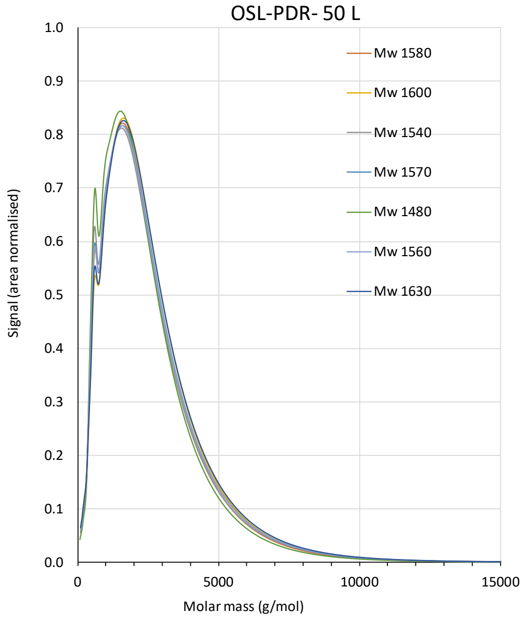

Figure S9. SEC curves of larger scale depolymerized OSL.

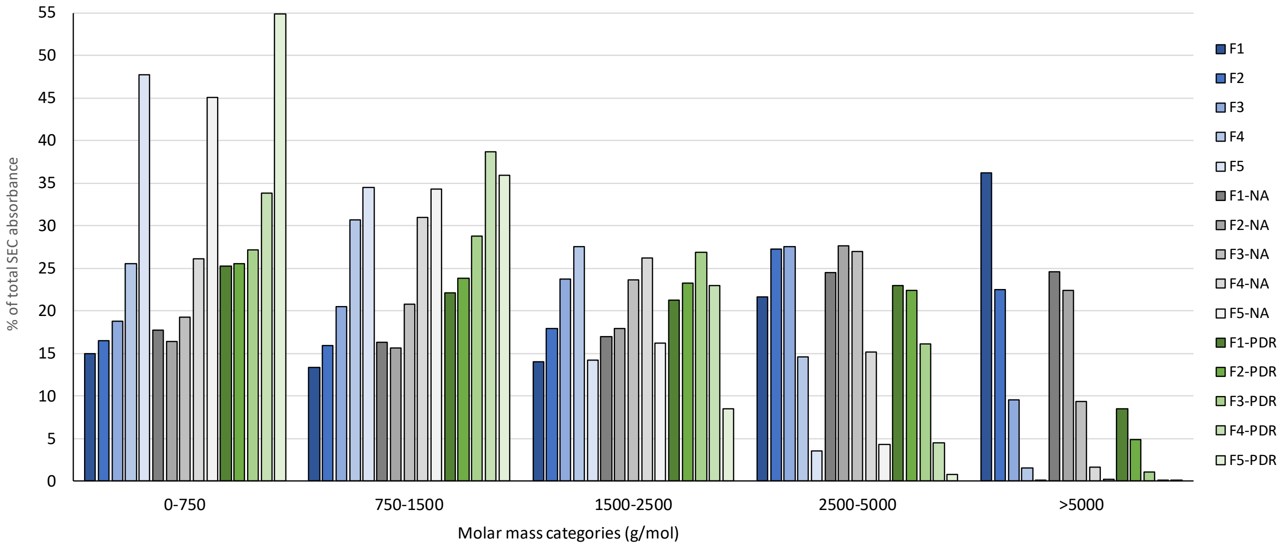

Figure S10. Molar mass distribution of isolated, non-aggregated (-NA) and partially depolymerized OSL fractions.

Table S3. Characteristics of depolymerized OSL fractions.

	F1	F2	F3	F4	F5
Molar mass (g/mol) [a]					
M_n	860	830	780	680	520
M_w	2170	1900	1530	1130	770
M_w/M_n	2.5	2.3	2.0	1.7	1.5
Hydroxyl groups content (mmol/g lignin) [b]					
Aliphatic OH	1.92 ± 0.08	1.93 ± 0.01	1.86 ± 0.12	2.02[c]	3.89 ± 0.04
5-substituted OH	2.29 ± 0.02	2.45 ± 0.03	2.52 ± 0.10	2.61	2.43 ± 0.16
G-OH	0.80 ± 0.00	0.82 ± 0.02	0.80 ± 0.04	0.78	0.74 ± 0.06
p-hydroxyphenyl OH	0.08 ± 0.01	0.08 ± 0.01	0.07 ± 0.00	0.06	0.06 ± 0.02
Total PhOH	3.16 ± 0.01	3.35 ± 0.05	3.39 ± 0.14	3.44	3.23 ± 0.23
Free COOH	0.02 ± 0.01	0.03 ± 0.00	0.02 ± 0.00	0.01	0.01 ± 0.00
COOH	0.08 ± 0.01	0.09 ± 0.01	0.09 ± 0.02	0.08	0.13 ± 0.00

[a] Determined by alkaline size exclusion chromatography. [b] Determined by ^{31}P NMR. [c] single ^{31}P NMR measurement.

Table S4. Characteristics of (larger scale depolymerized) OSL and mixture of high molar mass fractions.

	OSL	OSL-PDR	F1,2,3	F1,2,3-PDR
Molar mass (g/mol) [a]				
M_n	950	800	1090	730
M_w	2230	1580	3240	1790
M_w/M_n	2.4	2.0	3.0	2.7
Hydroxyl groups content (mmol/g lignin) [b]				
Aliphatic OH	1.89 ± 0.08	2.45 ± 0.07	1.86[c]	2.27 ± 0.07
5-substituted OH	2.40 ± 0.01	2.38 ± 0.03	2.21	2.61 ± 0.06
G-OH	0.77 ± 0.01	0.76 ± 0.01	0.74	0.87 ± 0.01
p-hydroxyphenyl OH	0.09 ± 0.01	0.07 ± 0.00	0.09	0.09 ± 0.01
Total PhOH	3.25 ± 0.02	3.22 ± 0.04	3.05	3.56 ± 0.06
Free COOH	0.01 ± 0.01	0.00 ± 0.00	0.03	0.01 ± 0.00
COOH	0.15 ± 0.00	0.15 ± 0.00	0.16	0.14 ± 0.01

[a] Determined by alkaline size exclusion chromatography. [b] Determined by ^{31}P NMR. [c] F1,2,3 hydroxyl group content was not analyzed but calculated from the fraction yield and fraction analysis shown in Table S2.

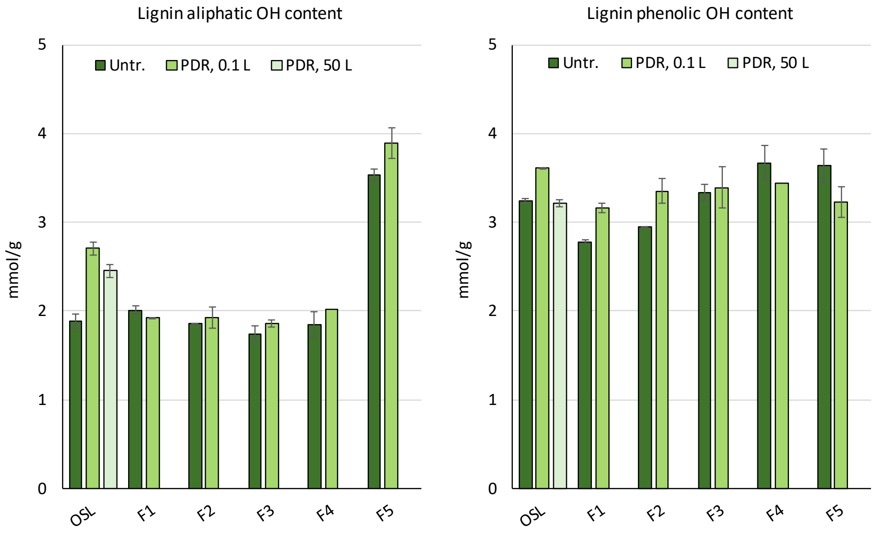

Figure S11. Aliphatic and total phenolic hydroxyl content of (depolymerized) OSL and its fractions.

Application of lignin in coatings

Experimental

Lignin-based PU coatings preparation

To assess how lignin molar mass, dispersity, hydroxyl group density and reactivity affect PU coating characteristics, a selection of lignins was made comprising OSL and lignin fraction F1, F4 and F5. Additionally, a mixture of F1, F2 and F3 (OSL-F123) was prepared to represent the higher molar mass fraction of the OSL. The selection also included the partially depolymerized OSL (OSL-PDR) and the partially depolymerized F123 (OSL-F123-PDR) mixtures.
Lignin-based PU coatings were synthesized by reacting the selected lignin fraction with an aliphatic polyisocyanate resin based on hexamethylene diisocyanate, namely, Desmodur® N3900 (from here on referred to as pHMDI), at different aliphatic OH/NCO ratios. As described in previous works [7-8], the desired amount of lignin and pHMDI were first dissolved in a suitable solvent at 30% wt concentration, under magnetic stirring, at room temperature for 1 h. To this end, various solvents were tested: tetrahydrofuran (THF), methyl ethyl ketone (MEK) and ethyl acetate (EA). Once lignin and pHMDI were completely dissolved, the solution was spin-cast on different substrates (glass, wood, aluminum, or steel) at 1200 rpm for 40 s by means of a WS-400-NPP spin processor (Laurell Technologies Corporation) and cross-linked in an oven for 1 h at 150 °C (coating thickness ~ 4 µm, as measured by surface profilometry). The completeness of the reaction between OH groups and NCO groups was assessed by Fourier-transform infrared (FTIR) spectroscopy. NCO groups were considered completely reacted when the N=C=O stretching signal at 2270 cm^{-1} in the FTIR spectrum disappeared. Then, the extent of the cross-linking reaction was also studied in terms of resistance to solvent washing, by dipping the obtained PU film in THF for 24 h and quantifying the extracted fraction by gravimetry. For each coating, the optimal lignin/pHMDI weight ratio (selected to be subsequently characterized) was considered the one in which all NCO groups were found to be reacted (from FTIR analysis) and that, at the same time, allowed the largest amount of lignin to be incorporated (i.e., lignin extracted by THF <10% wt).

Characterization of lignin-based PU coatings

Fourier-transform infrared spectroscopy (FT-IR) FT-IR spectroscopy was performed by means on a Nicolet Netxus 760 FTIR spectrophotometer. FTIR analyses were carried out on crosslinked PU coatings at increasing aliphatic-OH:NCO ratio, starting from 1:1, to assess the complete reaction of the isocyanate groups with the hydroxyls. Coatings to be analysed were deposed by spin-coating and then cured on a KBr disc. Spectra were recorded in transmission mode, at room temperature, in air, by accumulating 64 scans at a resolution of 4 cm^{-1} in the 4000-500 cm^{-1} wavenumber range.

Differential scanning calorimetry (DSC) DSC was employed to investigate the thermal transitions of lignins and the corresponding PU coatings. Measurements were carried out on 10-15 mg samples by means of a Mettler-Toledo DSC 823e instrument. Three runs (heating/cooling/heating) were performed: from 25 °C to 150 °C to remove residual solvent from coatings (or water from lignins); from 150°C to 25°C; and from 25°C to 200°C, all at a scan rate of 20 °C/min under nitrogen flux. The Tg of the samples was evaluated as the inflection point in the second heating run.

Optical contact angle measurements (OCA) Static optical contact angle measurements on the lignin-based PU films deposited on a glass substrate were performed using an OCA 20 (Data Physics) instrument equipped with a CCD camera and a 500 µL Hamilton syringe at room temperature. Around 15 measurements were performed in different regions of each PU coating. Ultrapure water and diiodomethane were used as probe liquids.

Atomic force microscopy (AFM) Nanoindentation measurements were performed by AFM with a NSCRIPTOR system driven by SPMCockpit software (NanoInk., Skokie, IL) instrument in ambient conditions (temperature in the 18−22 °C range and relative humidity between 25% and 40%) using commercially available silicon ACT probes purchased from AppNano (Santa Clara, CA) with a nominal spring constant of 37 N/m. The actual value of spring constants was calculated using the Sader approach.[9] The elastic modulus of the coatings was obtained from force−distance curve measurements based on the Sneddon model.[10] According to Sneddon, the relation between the deflection *d* and the indentation δ can be written as follows:

$$d = \frac{2}{\pi} \frac{E_s}{(1 - \nu_s^2)} \frac{\tan\theta}{k} \delta^2$$

where E_s is the elastic modulus of the coating (assuming an infinitely higher modulus of the probe compared to the sample) and ν_s its Poisson ratio, which was assumed to be equal to 0.35 for lignin-based PU film.[11] ϑ is the half-opening angle of the

silicon tip (~24° in our case, according to scanning electron microscopy images obtained after indentations) and k is the spring constant of the probe cantilever. The value of elastic modulus was calculated by interpolating the curves with the Sneddon model in the region of reversible elastic deformations according to the method developed in a previous paper.[12]

Pull-off adhesion tests The adhesion strength of the PU coatings on different substrates (namely, glass, wood, aluminum, and stainless steel) was evaluated by means of a ARW-T05 tester by measuring the pulling force required to detach a 20 mm diameter aluminum dolly adhered to the PU film through an epoxy adhesive (Araldite 2011, curing cycle: 24 h at 50 °C).

Results

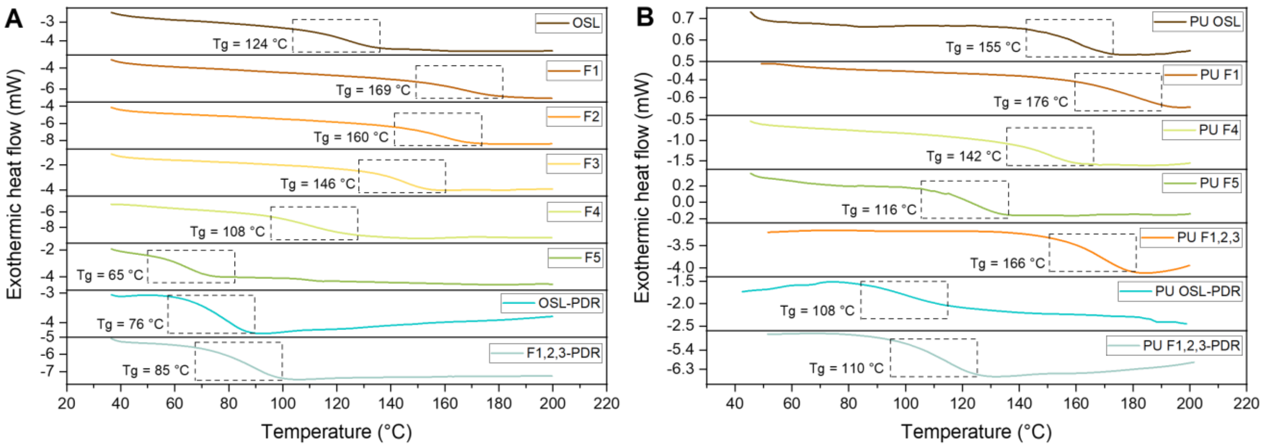

Figure S12. (A) DSC curves of pristine lignin (OSL), lignin fractions (F1-F5), depolymerized OSL (OSL-PDR) and depolymerized fraction mixture (F1,2,3-PDR); (B) DSC curves of the corresponding PU coatings.

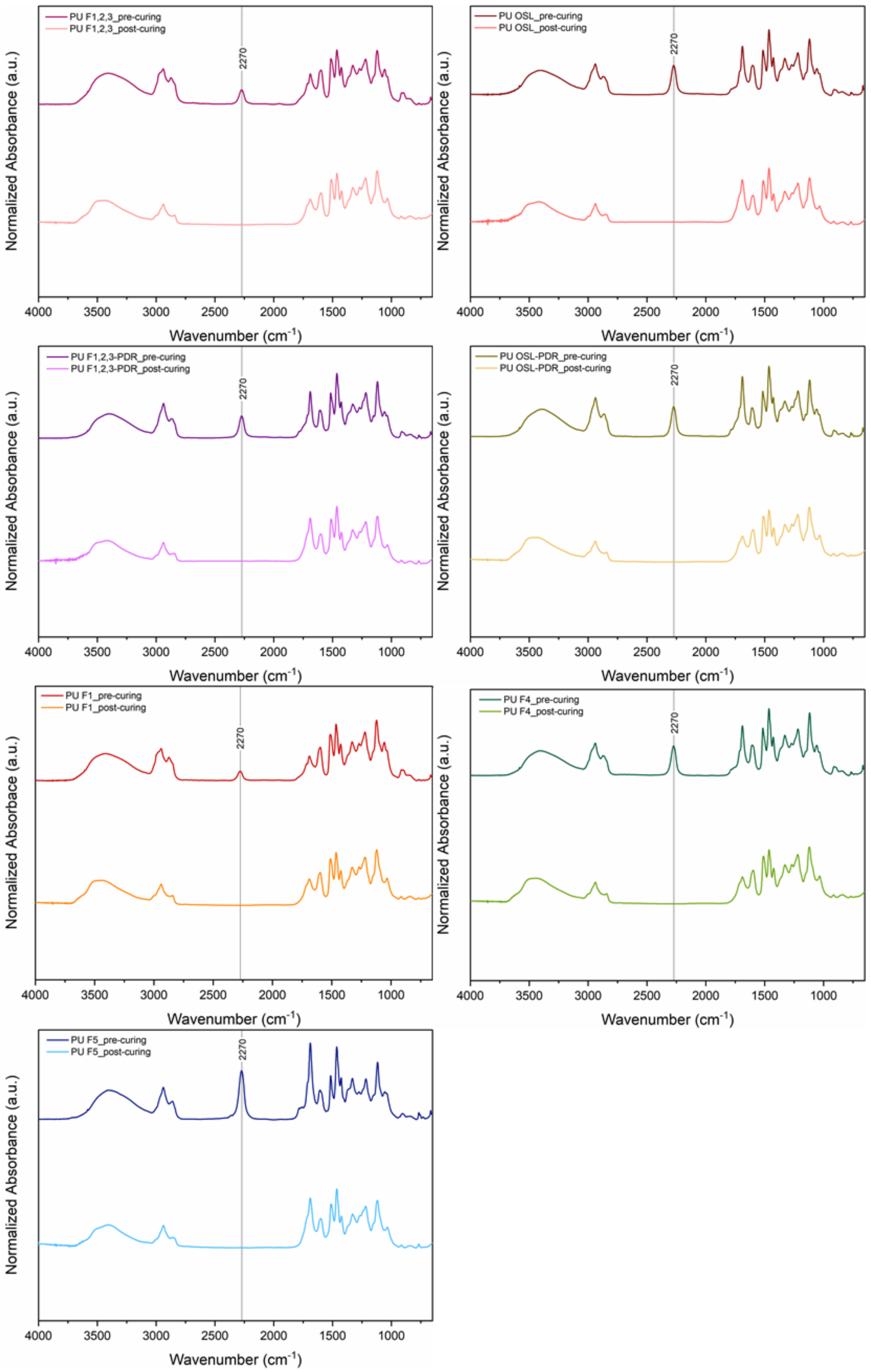

Figure S13. FTIR spectra of all lignin-based PU coating formulations before curing (pre-curing) and after curing (post-curing). The signal associated with N=C=O bond stretching vibration is highlighted (2270 cm^{-1}).

References

1. Smit, A. T.; Verges, M.; Schulze, P.; van Zomeren, A.; Lorenz, H., Laboratory-to Pilot-Scale Fractionation of Lignocellulosic Biomass Using an Acetone Organosolv Process. *ACS Sustain. Chem. Eng.* **2022**, 10 (32), 10503–10513.
2. Sluiter, J. B.; Ruiz, R. O.; Scarlata, C. J.; Sluiter, A. D.; Templeton, D. W., Compositional analysis of lignocellulosic feedstocks. 1. Review and description of methods. *J. Agric. Food Chem.* **2010,** 58 (16), 9043-9053.
3. Smit, A.; Huijgen, W., Effective fractionation of lignocellulose in herbaceous biomass and hardwood using a mild acetone organosolv process. *Green Chem.* **2017,** 19 (22), 5505-5514.
4. Smit, A. T.; van Zomeren, A.; Dussan, K.; Riddell, L. A.; Huijgen, W. J.; Dijkstra, J. W.; Bruijnincx, P. C., Biomass Pre-Extraction as a Versatile Strategy to Improve Biorefinery Feedstock Flexibility, Sugar Yields, and Lignin Purity. *ACS Sustain. Chem. Eng*. **2022**, 10, 6012-6022.
5. Sluiter, A.; Hames, B.; Ruiz, R.; Scarlata, C.; Sluiter, J.; Templeton, D., Determination of ash in biomass. *National Renewable Energy Laboratory* **2008,** (NREL/TP-510-42622).
6. Smit, A. T.; Dezaire, T.; Riddell, L. A.; Bruijnincx, P. C. A., Reductive Partial Depolymerization of Acetone Organosolv Lignin to Tailor Lignin Molar Mass, Dispersity and Reactivity for Polymer Applications. *ACS Sustain. Chem. Eng.* **2022**, accepted.
7. Griffini, G.; Passoni, V.; Suriano, R.; Levi, M.; Turri, S., Polyurethane coatings based on chemically unmodified fractionated lignin. *ACS Sustain. Chem. Eng.* **2015,** 3 (6), 1145-1154.
8. de Haro, J. C.; Allegretti, C.; Smit, A. T.; Turri, S.; D'Arrigo, P.; Griffini, G., Biobased polyurethane coatings with high biomass content: tailored properties by lignin selection. *ACS Sustain. Chem. Eng.* **2019,** 7 (13), 11700-11711.
9. Sader, J. E.; Sanelli, J. A.; Adamson, B. D.; Monty, J. P.; Wei, X.; Crawford, S. A.; Friend, J. R.; Marusic, I.; Mulvaney, P.; Bieske, E. J., Spring constant calibration of atomic force microscope cantilevers of arbitrary shape. *Rev. Sci. Instr.* **2012,** 83 (10), 103705.
10. Sneddon, I. N., The relation between load and penetration in the axisymmetric Boussinesq problem for a punch of arbitrary profile. *Int. J. Eng. Sci.* **1965,** 3 (1), 47-57.
11. Cousins, W.; Armstrong, R.; Robinson, W., Young's modulus of lignin from a continuous indentation test. *J. Mat. Sci.* **1975,** 10 (10), 1655-1658.
12. Suriano, R.; Credi, C.; Levi, M.; Turri, S., AFM nanoscale indentation in air of polymeric and hybrid materials with highly different stiffness. *Appl. Surf. Sci.* **2014,** 311, 558-566.
